# Unveiling the Potential Binding Targets of Celastrol in Colorectal Cancer: A Proteomic Profiling Approach Integrating Cellular Thermal Shift Assay and Pulse Proteolysis

**DOI:** 10.1021/acs.jproteome.4c00738

**Published:** 2025-06-06

**Authors:** Ti Lin, Shang-Lin Yang, Chao-Jung Chen, Pei-Fen Liu

**Affiliations:** † Department of Food Science and Biotechnology, 106252 National Chung Hsing University, Taichung City 402, Taiwan; ‡ Graduate Institute of Microbiology, College of Medicine, National Taiwan University, Taipei 100, Taiwan; § Department of Biochemistry and Molecular Biology, National Cheng Kung University, Tainan City 701, Taiwan; ∥ Graduate Institute of Integrated Medicine, 38019 China Medical University, Taichung City 402, Taiwan

**Keywords:** colorectal cancer, celastrol, target identification, thermal proteome profiling, pulse proteolysis

## Abstract

Celastrol, a natural compound classified as a pentacyclic triterpenoid, has demonstrated efficacy in inhibiting human colorectal cancer cells’ growth, adhesion, and metastasis through various signaling pathways. However, the specific protein target responsible for the effects of celastrol remains unclear, limiting its potential for further applications in colon cancer treatment and drug development. In this study, we propose a novel approach by combining a cellular thermal shift assay and pulse proteolysis techniques to identify the potential binding proteins of celastrol. Utilizing proteomic profiling on 2-dimensional electrophoresis, we successfully identified eight potential binding targets. MALDI-TOF mass spectrometry was conducted to verify these proteins as EEF2, STIP1, GAPDH, FLNA, SETSIP, GANAB, TXNDC17, and PRDX2. Our results provide valuable insights into the protein targets through which celastrol exerts its pharmacological effects, opening up new avenues for targeted therapies and drug development in colorectal cancer.

## Graphical abstract

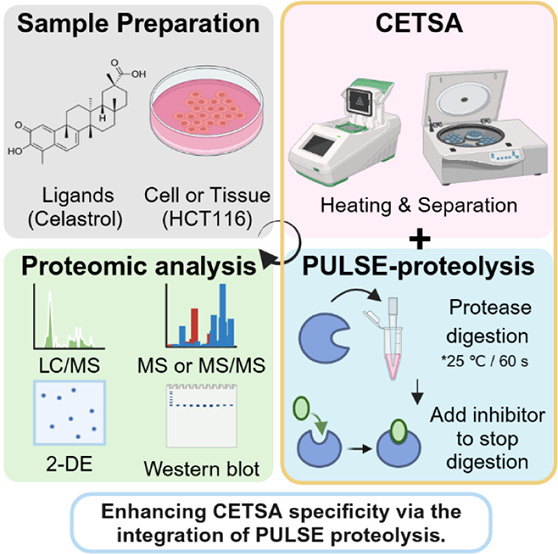

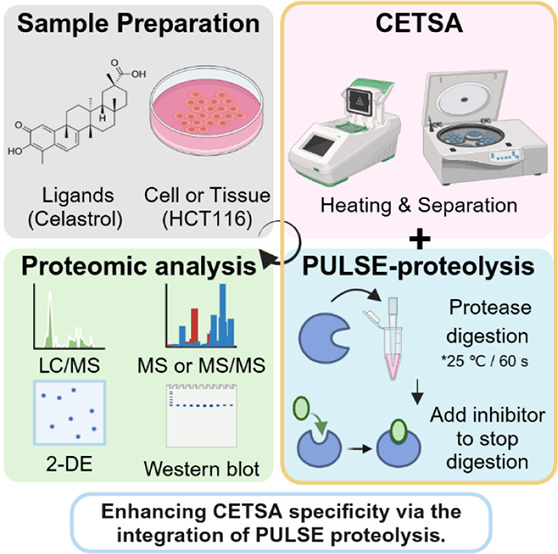

## Introduction

Colorectal cancer (CRC) is a highly prevalent and deadly malignancy, ranking as the third leading cause of cancer-related mortality in 2020, with approximately 1.9 million new cases reported.^1,2^ Chemotherapy has long been established as a therapeutic approach for CRC, with agents such as fluoropyrimidine (5-FU), oxaliplatin (OX), and irinotecan (IRI) commonly utilized. Combination regimens like FOLFOX (5-FU+OX) have shown improved overall survival rates, extending survival by nearly 20 months.^3^ Despite these advancements, CRC often develops mechanisms that confer resistance to chemotherapy, rendering the treatment ineffective.^4^ One such mechanism involves the overexpression of P-glycoprotein, an ATP-dependent membrane transporter that actively effluxes multiple drugs from cancer cells, thereby evading their cytotoxic and apoptotic effects. Therefore, there is a pressing need to explore novel and more efficacious compounds to enhance the treatment of colorectal cancer.

Celastrol, a natural bioactive compound derived from the root of *Hook F*, belongs to the triterpene family and exhibits remarkable therapeutic activities.^5,6^ It possesses several functional groups, including hydroxyl, carboxyl, and quinone methide groups, which are essential for its affinity and protein binding capabilities.^7^ Extensive research has implicated celastrol in diverse anticancer properties, such as antioxidant and anti-inflammatory effects,^8,9^ antiangiogenic actions,^10^ induction of apoptosis and autophagy,^11,12^ and cell cycle arrest.^13^ In the context of colorectal cancer (CRC), celastrol has been shown to modulate multiple signaling pathways.^14^ For example, it promotes β-catenin degradation through the HSF1–LKB1–AMPKα–YAP pathway,^15^ influences the TGF-β1/Smad signaling to modulate EMT expression,^16^ and activates the heat-shock response by inhibiting Hsp90 activity.^17,18^ Moreover, celastrol affects protein kinases involved in the Raf/MEK/ERK and PI3K/AKT/mTOR signaling pathways, thereby promoting apoptosis and autophagy.^19^ Although previous studies have reported some celastrol binding proteins, such as PRDX1, PRDX2, VAMP7, and RAB7,^20–22^ these targets alone cannot fully explain the therapeutic activity of celastrol. Therefore, we aim to comprehensively identify the potential cellular binding targets of celastrol using a proteomics profile approach.

Thermal proteome profiling is a proteomic technique that combines the cellular thermal shift assay (CETSA) with mass spectrometry-based analysis.^23^ CETSA exploits the principle that upon heating, proteins tend to unfold and become insoluble at a specific temperature.^24^ When a ligand binds to a protein, it alters the thermal stabilization of the target protein, leading to a shift in its melting temperature curve. This shift can be monitored using protein detection methods such as mass spectrometry and Western blotting. CETSA offers several advantages, including its compatibility with unpurified protein samples and applicability in live cells or tissues without the need for expensive fluorescent dyes or isotope calibrations.^25^ Thus, the CETSA is a convenient approach to study drug-target interactions. However, the CETSA has certain limitations that may influence the obtained results. For example, some proteins may have multiple domains, and the ligand might specifically bind to a domain that does not globally affect the thermal stability of the entire protein.^26,27^ Additionally, certain proteins can remain in partially unfolded states after heat treatment and other ligands may alter solution properties such as pH, ionic strength, or solvent additives, potentially affecting protein solubility.^28^ To address these potential confounding factors, we employed pulse proteolysis in conjunction with the CETSA.

Pulse proteolysis is a valuable quantitative tool for assessing protein stability under different conditions.^29^ It provides insights into the unfolding and folding states of proteins and indirectly explores protein–ligand interactions. The underlying principle of pulse proteolysis relies on the fact that when a protein is either fully or partially unfolded, a high concentration of protease can rapidly hydrolyze the protein. In contrast, if the protein remains in a fully folded state, then it can resist proteolytic digestion. By leveraging these characteristics, we can investigate whether the unfolding equilibrium constant of a protein changes due to interactions with a ligand. The combination of the CETSA with pulse proteolysis offers several advantages. The use of a high concentration of protease immediately following heat treatment ensures that any proteins denatured by heat are completely hydrolyzed. Furthermore, this approach enhances the detectability of proteins that may not exhibit significant changes in classic CETSA experiments.

In this study, we introduced pulse proteolysis as a novel addition to the Cellular Thermal Shift Assay (CETSA or TPP) for the first time, aiming to analyze the potential target proteins of celastrol. Through the integration of CETSA-PULSE, we successfully identified eight candidate proteins, namely, EEF2, STIP1, GAPDH, FLNA, SETSIP, GANAB, TXNDC17, and PRDX2. These proteins hold promise in unraveling the detailed mechanisms underlying celastrol’s actions in colorectal cancer cells and are expected to provide crucial insights for future drug design endeavors. Moreover, the CETSA-PULSE approach offers a valuable alternative for researchers investigating compound-protein interactions, expanding the available options in this domain of scientific inquiry.

## Material and Methods

### Chemical and Reagents

Celastrol was purchased from Toronto Research Chemicals. Thermolysin was purchased from Sigma-Aldrich. A 2 mL Zeba Spin Desalting Column was purchased from Thermo Scientific. 2-D Quant Kit was purchased from GE Healthcare Life Sciences. Cell counting kit-8 was purchased from TargertMol. The primary antibodies GAPDH, EEF2, FLNA, and STIP1 were purchased from Santa Cruz. The elute solution (97.5% ACN, 2.5% trifluoroacetic acid (TFA)) was used in Gel-LC-MS/MS. The rehydration buffer (8 M urea, 2% CHAPS, 0.5% (v/v) ampholytes 3–10, and 0.001% bromophenol blue) was used in two-dimensional gel electrophoresis (2DE). The DTT equilibration solution (100 mM DTT dissolved in SDS equilibration buffer) was used in 2DE. The IAA equilibration solution (250 mM iodoacetamide dissolved in SDS equilibration buffer) was used in 2DE. The stacking gel (0.5% agarose in 1 × running buffer) was used in 2DE. The matrix solution (1–1.5 mg MALDI Matrix dissolved in 1 mL TA buffer (66.7% ACN, 3.3% μL TFA (1%), 30% ddH2O)) was used in MALDI-TOF/TOF.

### Instrument

NanoLC-MS/MS was performed with a nanoflow ultraperformance liquid chromatography system (UltiMate 3000 RSLCnano system; Dionex) coupled to a hybrid quadrupole time-of-flight (Q-TOF) mass spectrometer (maXis Impact; Bruker). Peptides trap column (Acclaim PepMap C18, 5 μm, 100 Å, 20 μm × 100 mm; Thermo Scientific). Peptides analytical column (Acclaim PepMap C18, 2 μm, 100 Å, 75 μm × 250 mm; Thermo Scientific). Desalting column (Thermo Scientific). Immobiline DryStrip pH3–10, 13 cm, linear (GE Healthcare), and IPGphor 3 (GE Healthcare) was used in IEF. MALDI-TOF/TOF target plate (384 well AnchorChip). TOF/TOF mass spectrometry (Ultraflex III).

### Cell Culture

HCT116 cells were obtained from the Bioresource Collection and Research Center in Taiwan. HCT116 cells were cultured in McCoy’s 5A medium containing 10% fetal bovine serum, penicillin (100 U/mL), and streptomycin (100 μg/mL) at 37 °C in a humidified atmosphere with 5% CO_2_.

### Cell Viability Assay

The cytotoxicity of celastrol was assessed by the CCK-8 assay. Cells were seeded in a 96-well culture plate with a density of 4000 cells per well and treated with celastrol for 72 h. After that, the CCK-8 reagent was diluted with McCoy’s 5A medium (medium: CCK-8 = 10:1), and the cells were incubated in the diluted CCK-8 solution for 4 h. We used the iMark Microplate Absorbance Reader from Bio-Rad to test the OD values at 450 nm.

### Cellular Thermal Shift Assay (CETSA)

CETSA experiment was carried out as previously described.^25^ In brief, cells were harvested with a cell density of 1.3 × 10^7^ cells/mL and were lysed by freeze–thawing with liquid nitrogen 3–5 times. After 13,800 *g* centrifugation, the HCT116 cell suspension was incubated with DMSO (control) or celastrol (100 μM) for 15 min at 37 °C. Then, we dispensed two group samples into each 100 μL aliquot. Each aliquot was heated to a different temperature and then incubated at room temperature. The heat-treated samples were subjected to 20,000 *g* centrifugation to remove the conformational-loss proteins. Finally, the samples were analyzed by using proteomic methods.

### CETSA-Pulse Proteolysis

The procedure is the same as the CETSA experiment. The only difference is that after centrifuging the heat-treated samples, Thermolysin was added into the samples to a final concentration of 0.20 mg/mL. After the samples were incubated for 1 min at 25 °C, we stopped the proteolysis reaction by adding ethylenediaminetetraacetic acid (EDTA) in an equal volume of Thermolysin.

### One-Pot Analysis

Here, we use one-pot analysis as previously reported to save time.^30^ We took an equal volume of the sample at each temperature and mixed all of the samples into a single sample. The mixed sample was used for proteomic analysis to increase the efficiency of the protein screening.

### Isothermal Dose Response Fingerprinting (ITDRF)

An ITDRF experiment was performed as previously described.^25^ In short, after freeze–thawing and centrifugation, cell suspensions were incubated with various concentrations of celastrol ranging from 5 to 1280 μM for 15 min at 37 °C. The reacted cells were heated at a given temperature and then collected by centrifugation. Finally, samples were analyzed by using proteomic methods.

### Gel-LC-MS/MS

Samples were separated by SDS gel electrophoresis and stained with a Coomassie Brilliant Blue *R*-250. The specific bands were cut into 1.0 mm slices and placed in 0.6 mL tubes. For cleaning, tubes were shaken in 200 μL ddH_2_O for 15 min, then replaced with 200 μL NH_4_HCO_3_/ACN (50 mM) and shaken for 15 min. Subsequently, samples were immersed in 200 μL of ACN (5 min) before drying in a speed vacuum. For reduction and alkylation, samples were incubated with 200 μL of dithiothreitol (DTT) (100 mM) for 45 min at 56 °C. After removing DTT, 200 μL of iodoacetamide (IAA) (55 mM) is added and reacted in the dark for 30 min at room temperature. Tubes are washed again with 200 μL of NH_4_HCO_3_/ACN (50 mM) for 10 min, followed by 200 μL of ACN for 5 min and drying in a speed vacuum. For digestion, trypsin stock solution (20 ng/mL) is diluted 1:50 with ammonium bicarbonate (50 mM) and ddH_2_O. Then, 20 μL of diluted trypsin was added and reacted for 30 min at 4 °C. Next, 20 μL NH_4_HCO_3_ (50 mM) is added and incubated for 13–14 h at 37 °C. Subsequently, each tube was treated with a 2 μL elute solution and shaken with ultrasonicator for 10 min, repeating this step once. Supernatants were collected in new tubes that may contain peptides that have diffused out of the spot. Extracts are dried in a Speed Vac and analyzed by NanoLC-MS/MS which details listed in Supporting Information.

After loading the sample, the peptides were eluted from a trap column into an analytical column connected to a nanoelectrospray ionization source on the Q-TOF mass spectrometer.

The proteins were identified using FlexAnalysis 3.0 software by searching the SwissProt-Protein sequence database. The details of FlexAnalysis 3.0 software parameters for identified proteins of Gel-LC-MS/MS were listed in Supporting Information.

### Western Blot

The samples were separated by 12% SDS-PAGE and then transferred to a PVDF membrane. The membrane was blocked with 5% skim milk for 1 h at room temperature, 55 rpm. After washing, it was incubated with primary antibodies overnight at 4 °C. The membrane was subsequently washed and immersed in the secondary antibodies for 1 h at room temperature at 60 rpm. Finally, the antigen was detected with an enhanced chemiluminescence (ECL) substrate solution. The photo was taken by BioSpectrum Imaging System.

### Two-Dimensional Gel Electrophoresis (2DE)

To remove excessive salts in samples, we used the desalting column. 300 μg of the protein sample was concentrated with the SpeedVac evaporator to reach a total volume of 125 μL. The concentrated samples were mixed with 125 μL of rehydration buffer and separated by isoelectric focusing (IEF). After IEF, the strips were slightly washed with running buffer and immersed in DTT equilibration solution for 15–20 min. Then, the strips were transferred to an IAA equilibration solution. The strips were put on the 12.5% polyacrylamide gel of the second direction and sealed with a stacking gel. The electrophoresis program was listed in Supporting Information.

We picked up the spots with a *p* < 0.05 and a more than 1.3-fold change in relative intensity. The spot cleaning and digestion process is the same as that in Gel-LC-MS/MS. Finally, extracts were slightly dried in Speed Vac for 3 min and analyzed by MALDI-TOF/TOF.

### MALDI-TOF/TOF

0.2 μL aliquot of matrix solution was spotted onto a target plate (600 μM) and dried. 0.5 μL of peptide extraction was dropped on the dried matrix solution and waited for the second layer to dry. 0.2 μL of matrix solution was spotted again on the dried peptide extraction and waited for dry.

The mass spectrometer equipped with a delayed extraction ion source device was first calibrated with Bradykinin 1–7 ([M + H]+ 757.39), Angiotensin II ([M + H]+ 1046.54), Angiotensin I ([M + H]+ 1296.68), Substance P ([M + H]+ 1374.74), Bombesin ([M + H]+ 1619.82), ACTH clip 1–17 ([M + H]+ 2093.09), ACTH clip 18–39 ([M + H]+ 2465) and Somatostatin28 ([M + H]+ 3147.4 to keep the error within ± 20 ppm. Next, the target plate was placed into the mass spectrometer. In Lift mode, we took the top 4 precursor ions of the signal intensity in the Peptide Mass Fingerprint (PMF) spectrum for fragmentation. We selected the generated precursor ions and fragment ions in the second field-free region to accelerate the separation in a two-stage gridless reflectron.

Finally, the data was analyzed with FlexAnalysis 3.0 software by searching the SwissProt-Protein sequence database. The details of FlexAnalysis 3.0 software parameters for identified proteins of MALDI-TOF/TOF were listed in Supporting Information.

### Statistical Analysis

I_37_ and *T*_m_ values were calculated by GraphPad Prism9 software using a variable logistic curve. The Melanie 8 was used to quantify each protein spot on the 2D gel. An unpaired Student’s *t* test was used to calculate *p* values using a statistics analysis system (SAS) or GraphPad Prism9.

The Boltzmann sigmoidal eq (eq 1) was employed to calculate the protein thermal melting curve (except for the thermal melting curve of FLNA 250 kDa).

1 $$Y=\frac{I_{37}}{1+\exp\left(\frac{T_{m}-X}{\mathrm{Slope}}\right)}$$

I_37_ is the relative intensity of initial protein residue levels at 37 °C, *T*_m_ is the temperature of half I_37_, and Slope is the relationship between temperature and Gibbs free energy.

The Boltzmann sigmoidal eq (eq 2) was employed to calculate the protein thermal melting curve of FLNA 250 kDa, as the protein residue level increases with elevated temperature in the range of 37–49 °C.

2 $$Y=\frac{Y_{\mathrm{TOP}}}{1+\exp\left(\frac{T_{m}-X}{\mathrm{Slope}}\right)}$$

Y_TOP_ is calculated by the formula (Y_TOP_=a*X+b), *T*_m_ is the temperature of half I_37_, and Slope is the relationship between temperature and Gibbs free energy.

The Boltzmann sigmoidal eq (eq 3) was employed to calculate the dose-dependent curve (except for the thermal melting curve of FLNA 250 kDa).

3 $$Y=\frac{I_{0}}{1+\exp\left(\frac{{\mathrm{IC}}_{50}-X}{\mathrm{Slope}}\right)}$$

I_0_ is the relative intensity of initial protein residue levels at 0 μM celastrol, and Slope is the relationship between ligand concentration and Gibbs free energy (M).

## Result and Discussion

### CETSA-Pulse is a Novel Alternative for Target Identification

The Cellular Thermal Shift Assay (CETSA), a recent advancement facilitating exploration into ligand-protein interactions within lysates, live cells, and even tissue samples, has been instrumental in identifying potential ligand binding targets.^25,31^ Researchers typically use significantly higher concentration (10–100 times higher than the IC_50_) to monitor the binding effect of ligands, as elucidated in the previous description.^24,25^ Our study investigated the impact of celastrol on HCT116 using the CCK-8 assay, employing a concentration of 100 μM celastrol (Figure S1). However, a limitation arises in delineating the targets interacting with celastrol via CETSA. Our findings revealed a general increase in the relative intensity of residual proteins in the celastrol-incubated group (Figure S3A-C), indicative of a “nonspecific” effect between celastrol and proteins. Notably, at temperatures exceeding 58 °C, this effect became pronounced. Literature suggests several extrinsic factors influencing protein solubility, encompassing pH, ionic strength, temperature, and solvent additives.^32,33^ Our inference suggests that the observed “nonspecific” effect may stem from the heightened water solubility of unfolded proteins upon incubation with celastrol, potentially impeding their removal via centrifugation.

Hence, our primary objective lies in devising a more effective methodology to eliminate proteins that undergo conformational alterations during the CETSA process. Our strategy enhanced CETSA by integrating PULSE proteolysis, leveraging its rapid protease action to degrade denatured proteins (Figure 1A). In contrast to traditional

**Figure 1:**
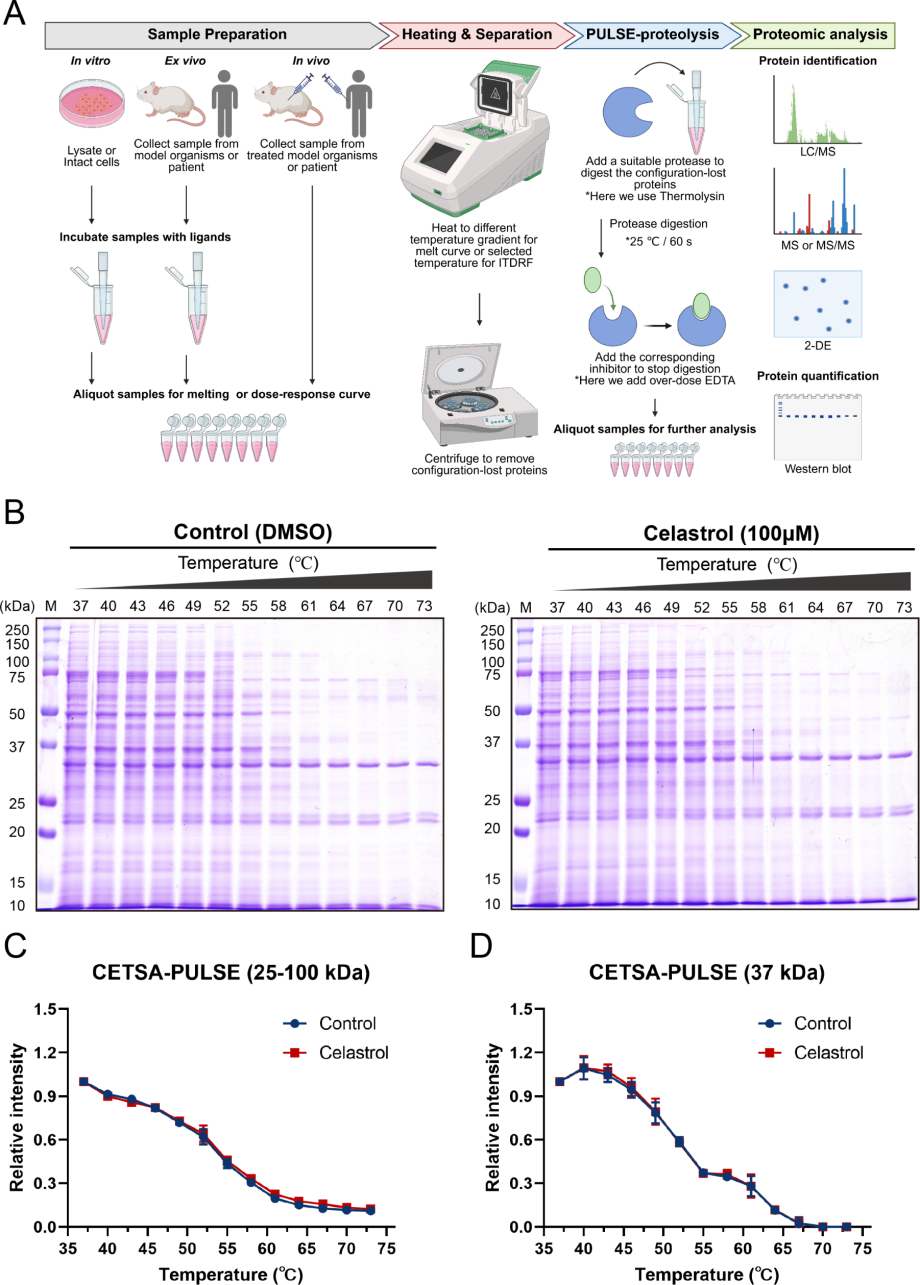
Integrating CETSA and Pulse-proteolysis to unveil the potential binding targets of celastrol. (**A**) Schematic overview of the CETSA-PULSE approach. In this study, our sample is prepared from the HCT116 cell line. According to classical CETSA protocols, samples can also be obtained from animal or human specimens.^24^ (**B**) The samples of the control group (DMSO) and the experimental group (100 μM celastrol) were detected by SDS-PAGE, and the 25–100 and 37 kDa bands were quantified. The other replicates are shown in Figure S2. The melting curves of (**C**) bands 25–100 and (**D**) 37 kDa do not show significant thermal shifts between the two groups. Results are plotted as average ± S.D., *p < 0.05, **p < 0.01, ***p < 0.001 using an unpaired two-tailed Student’s *t* test.

CETSA, CETSA-PULSE employs both physical and biochemical mechanisms to eliminate denatured proteins, extending its applicability across various ligands. Through protease-mediated digestion, we achieve more efficient removal of proteins experiencing conformational changes upon heating. This crucial attribute facilitates the exploration of potential binding proteins for diverse ligands in vitro while circumventing the “nonspecific” effects observed with certain compounds like celastrol. Furthermore, CETSA-PULSE retains the fundamental advantages of CETSA and extends its utility to investigate ligand-target protein interactions within cells or tissues. Nonetheless, an inherent limitation of CETSA-PULSE lies in the potential disappearance of specific target proteins due to protease digestion. To mitigate this concern, we opted for Thermolysin, given its tendency to hydrolyze hydrophobic residue sites less prominently across most conformational proteins.^29,32,34^ Nonetheless, there remains a possibility that certain celastrol-binding proteins could prove sensitive to Thermolysin action, posing challenges for monitoring via CETSA-PULSE. Despite this limitation, our findings unequivocally demonstrate the efficacy of CETSA-PULSE in averting the “nonspecific” effects induced by celastrol (Figures 1B and S2A,B). Here, we selected to quantify all bands within the 25–100 kDa range, as this interval covers most proteins or protein fragments. Also, we used the 37 kDa band as a single-band representative due to its clear and prominent appearance, facilitating more reliable quantification. The relative intensity observed at 25–100 kDa and 37 kDa signifies minimal impact on most protein residues in the presence of celastrol (Figure 1C,D), implying selective interactions with specific proteins.

### CETSA-Pulse is Reliable to Investigate Potential Binding Targets of Celastrol

Interestingly, Figure 1B illustrates a conspicuous reduction in protein residues within the 250–150 kDa range on the SDS PAGE gel in the presence of celastrol, indicating a potential interaction target (Figures 1B and 2A). To delve into this further, LC/MS analysis specifically targeting proteins within this molecular weight bracket revealed three candidates: Filamin-A (FLNA), Clathrin heavy chain 1 (CLH1), and Spectrin alpha chain, nonerythrocytic 1 (SPTN1) (Table 1). Notably, Filamin-A (FLNA) emerged with the highest Mascot Probability Based Scoring, prompting a prioritized investigation into its stability under celastrol influence. Our exploration via Western blotting unveiled destabilization of FLNA and its thermolysin-digested fragments upon exposure to celastrol (Figures 2B,C, and S3A). Utilizing the Boltzmann sigmoidal equation, we calculated the I_37_ and *T*_m_ values for various experimental groups. Although statistical analysis revealed no significant change in the *T*_m_ value of FLNA following incubation with celastrol, both FLNA and its fragment demonstrated a significant decrease in I_37_ after incubation with celastrol. These results suggest that the conformational changes in FLNA induced by celastrol may commence at temperatures approximating physiological conditions (Tables 2 and 3). Furthermore, employing Isothermal Dose–response Fingerprinting CETSA-PULSE (ITDRF_CETSA‑PULSE_) substantiated that concentrations of celastrol exceeding 80 μM notably reduced FLNA residue levels (Figures 2D and S3B). This comprehensive assessment confirms the applicability of CETSA-PULSE in identifying potential celastrol-binding proteins, prominently featuring FLNA among them.

**Figure 2:**
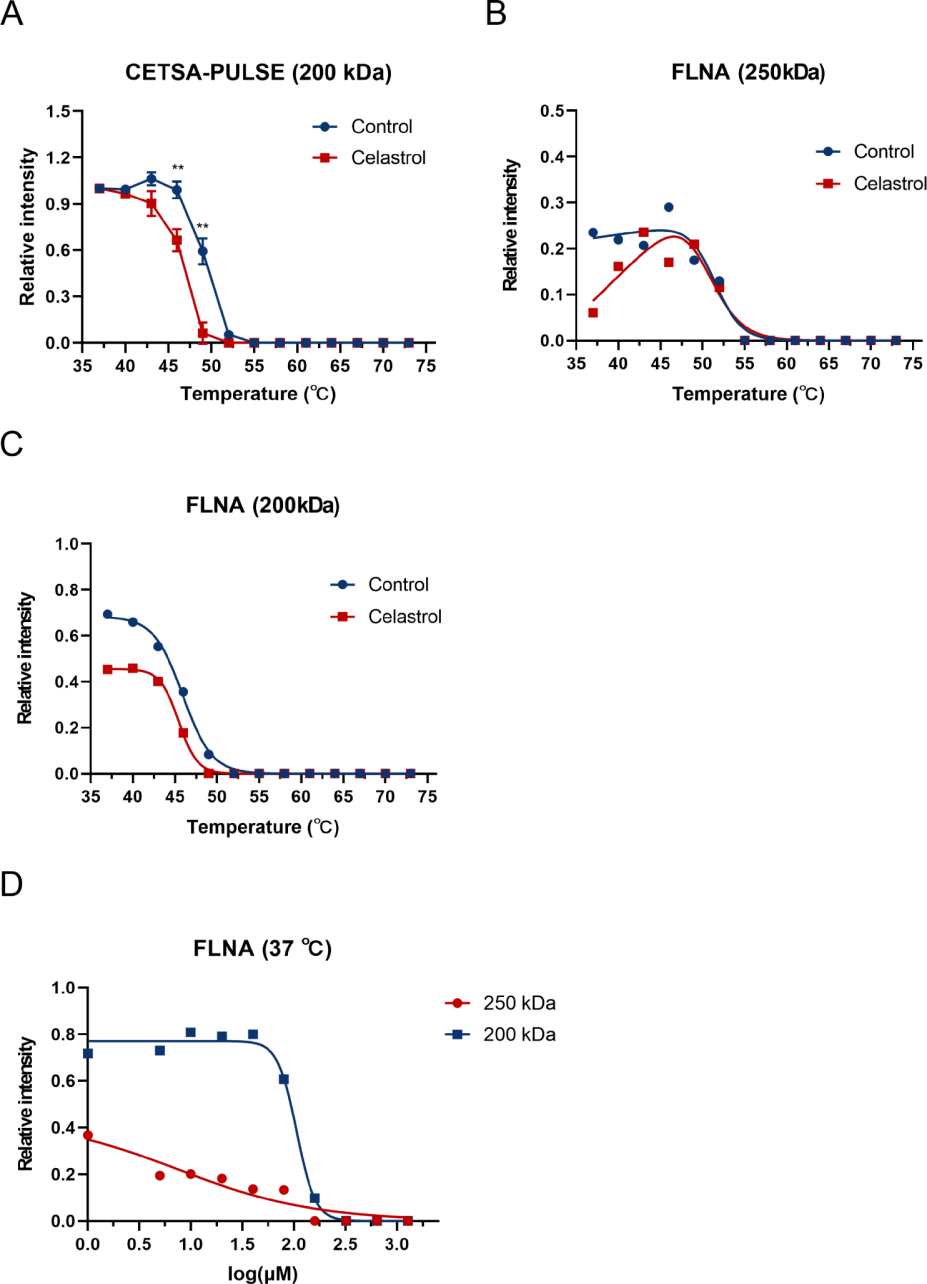
Identification of FLNA as a potential binding target of celastrol via the CETSA-PULSE. (**A**) The samples of the control group (DMSO) and the experimental group (100 μM celastrol) were detected by SDS-PAGE, and the melting curves of the bands (200 kDa) show thermal shifts between the two groups. Results are plotted as average ± S.D., *p < 0.05, **p < 0.01, ***p < 0.001 using an unpaired two-tailed Student’s *t* test. (**B**) The melting curves of FLNA (250 kDa) and (**C**) FLNA fragment 1 (∼200 kDa) show thermal shifts between the two groups. The *R* ^*2*^ of the FLNA (250 kDa) control group is 0.9603, and that of the celastrol group is 0.9262. The *R* ^*2*^ of the FLNA fragment 1 (∼200 kDa) control group is 0.9979, and the celastrol group is 0.9989. (**D**) Dose–response curves (250 and ∼200 kDa) show that incubation of samples with celastrol reduces FLNA levels at 37 °C. The *R* ^*2*^ of FLNA (250 kDa) is 0.9046, and FLNA fragment 1 (∼200 kDa) is 0.9939.

**Table 1:** Result of the LC/MS Analysis

Row^a^	1	2	3
**Protein identity**	Filamin-A	Clathrin heavy chain 1	Spectrin alpha chain, nonerythrocytic 1
**Gene symbol**	FLNA	CLH1	SPTN1
**GenBank accession** ^b^	3 × 10^08^	2 × 10^06^	9 × 10^07^
**Theoretical MW (kDa)/pI**	280.6/5.7	191.5/5.4	284.4/5.1
**Scores**	2695.9	1905.9	1519.8
**Peptides**	36	31	29
**SC [%]** ^c^	18.7	20.1	12.2

a The row numbers represent the top three protein scores identified by LC-MS.

b Accession numbers are based on NCBI database.

c Spectral counting (SC) counts the number of spectra identified for a given peptide in different biological samples and then integrates the results for all measured peptides of the protein(s) that are quantified.

**Table 2:** Statistical Analysis of Each Protein Group I_37_ Value^a^ ^b^

	Control		Celastrol		
Protein (kDa)	I_37_	95% CI	I_37_	95% CI	*p* value
FLNA (250 kDa)	0.223	0.188 to 0.270	0.086	0.067 to 0.144	*p* < 0.0001
FLNA (∼200 kDa)	0.684	0.657 to 0.712	0.455	0.444 to 0.467	*p* < 0.0001
STIP1 (63 kDa)	0.571	0.502 to 0.673	N/A	N/A	*p* < 0.0001
STIP1 (∼55 kDa)	0.481	0.424 to 0.583	0.146	0.130 to 0.167	*p* < 0.0001
STIP1 (∼50 kDa)	1.025	0.993 to 1.057	1.023	0.921 to 1.157	*p* = 0.9722
STIP1 (∼40 kDa)	0.253	0.226 to 0.293	N/A	N/A	*p* < 0.0001
GAPDH (37 kDa)	1.092	1.004 to 1.185	1.579	1.480 to 1.681	*p* < 0.0001
EEF2 (93 kDa)	0.065	0.048 to 0.085	0.318	0.280 to 0.370	*p* < 0.0001
EEF2 (∼37 kDa)	0.029	0.022 to 0.037	0.190	0.179 to 0.202	*p* < 0.0001
EEF2 (∼30 kDa)	0.923	0.889 to 0.958	0.676	0.635 to 0.718	*p* < 0.0001

a 1 N/A means the relative intensity of the protein is 0 and the value obtained by the equation is unstable.

b An unpaired two-tailed Student’s t-test is used to analyze. *p < 0.05, **p < 0.01, ***p < 0.001, ****p < 0.0001.

**Table 3:** Statistical Analysis of Each Protein Group *T*_m_ Value^a^ ^b^

	Control		Celastrol		
Protein (kDa)	*T*_m_	95% CI	*T*_m_	95% CI	*p* value
FLNA (250 kDa)	51.4	48.5 to 53.8	50.2	39.6 to 52.8	*p* = 0.7139
FLNA (∼200 kDa)	45.9	45.6 to 46.3	45.4	45.3 to 45.6	*p* = 0.0142
STIP1 (63 kDa)	43.3	42.4 to 44.3	N/A	N/A	N/A
STIP1 (∼55 kDa)	46.6	44.5 to 47.9	44.4	43.3 to 45.3	*p* = 0.0279
STIP1 (∼50 kDa)	54.4	54.0 to 54.8	50.3	48.7 to 51.7	*p* < 0.0001
STIP1 (∼40 kDa)	52.6	50.5 to 54.3	N/A	N/A	N/A
GAPDH (37 kDa)	58.0	56.9 to 58.9	58.1	57.2 to 58.8	*p* = 0.9193
EEF2 (93 kDa)	55.3	51.7 to 58.6	48.5	46.8 to 50.0	*p* = 0.0011
EEF2 (∼37 kDa)	59.3	57.4 to 62.2	58.1	57.5 to 58.7	*p* = 0.3301
EEF2 (∼30 kDa)	58.2	58.0 to 58.6	58.1	57.7 to 58.9	*p* = 0.7402

a 1 N/A means the relative intensity of the protein is 0 and the value obtained by the equation is unstable.

b An unpaired two-tailed Student’s t-test is used to analyze. *p < 0.05, **p < 0.01, ***p < 0.001, ****p < 0.0001.

### Celastrol Potentially Interacts Eight Different Proteins in Colorectal Cancer Cells

In our pursuit to delve deeper into celastrol-binding proteins, we employed a synergistic approach by integrating two-dimensional (2D) gel electrophoresis with a streamlined one-pot analysis method, aiming to streamline the protein stability assessment. Leveraging Melanie 8 software, we effectively mitigated staining and displacement discrepancies among protein spots, facilitating the quantification of relative intensities for each spot on the 2D gel (Figures 3 and S5A,B). Subsequent statistical analysis utilizing SAS software, specifically employing Student’s *t* test, discerned nine protein spots exhibiting significant abundance alterations (*p* < 0.05). Notably, these changes surpassed an abundance ratio threshold of 1.3, reinforcing their substantial divergence. Under the influence of celastrol, three protein spots exhibited augmented relative intensities, while six other protein spots showcased a decrease in relative intensity (Table S1). This comprehensive analysis delineates the distinct impact of celastrol on specific protein profiles, shedding light on potential targets and alterations within the proteomic landscape.

**Figure 3:**
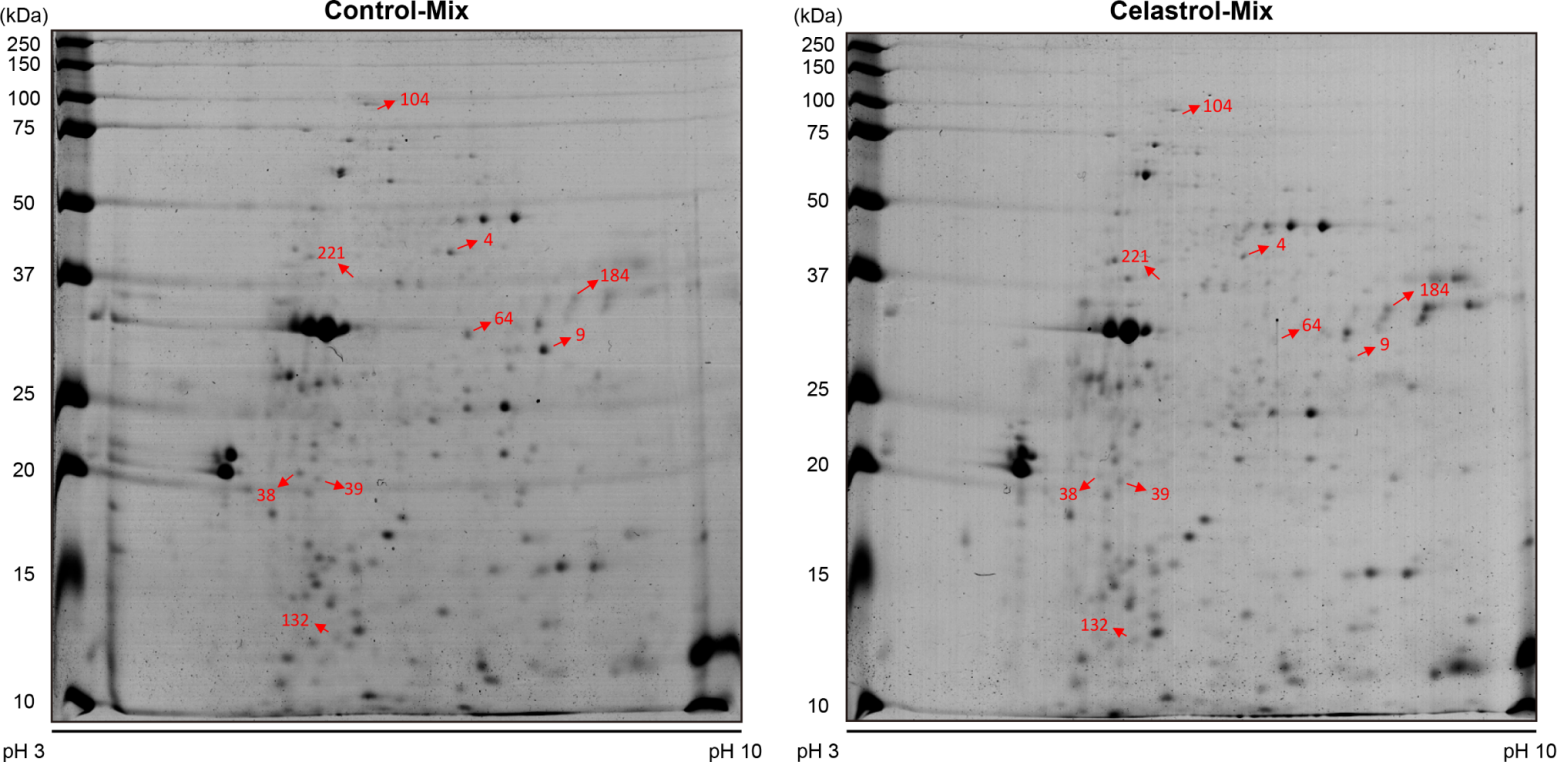
Celastrol potentially interacts with nine different spots on 2D gel. Nine different spots show significant level differences on the 2D gel. One representative 2D gel is shown here. The other replicates are shown in Figure S5. Statistical analysis by SAS software and Student’s *t* test (*p* < 0.05) are shown in Table S1.

Following this, nine protein spots with notable distinctions underwent analysis utilizing a MALDI-TOF/TOF mass spectrometer for precise protein identification via a Mascot database alignment. Identification scores surpassing the established threshold indicated successful matches, while “NS” denoted a lack of significant correspondence in the database. From the TOF results, eight protein spots exceeded their respective threshold scores, revealing the identities of seven proteins: EEF2, STIP1, GAPDH, FLNA, SETSIP, GANAB, and PRDX2. Notably, discrepancies in the positions of certain protein spots on the 2D gel compared with their original size (kDa) were observed. This characteristic observed in the results aligns with a trait of CETSA-PULSE, wherein residue differences detected in proteomic analysis may stem from protein fragments rather than intact proteins. Additionally, while one protein spot failed to meet the individual threshold score, the TOF/TOF results confirmed its alignment with the threshold score, conclusively identifying the protein TXNDC17.

Theoretical Mr/pI values represent a protein’s anticipated molecular weight and isoelectric point in the database. Variations between the theoretical pI value of each protein and its positioning on the 2D gel arise due to protease-induced modifications altering the protein properties. Peptide Mass Fingerprinting (PMF) quantifies the ratio of matched peptides to the full length of protein peptides obtained from MALDI-TOF analysis. However, only the TXNDC17 protein lacked PMF data due to the absence of a TOF score. Collectively, this rigorous analysis unveiled eight distinct proteins affected by celastrol in colorectal cancer cells (Table S2).

Next, we performed protein annotation through UniProt to identify their involvement in biological processes, cellular component distribution, and molecular functions (Table 4). Within the cohort of eight candidates, PRDX2 emerged as a direct target of celastrol in gastric cancer cells.^22^ Recent investigations have underscored celastrol’s CRC-suppressive effects via covalent targeting of PRDX1 and PRDX2.^20^ Both PRDX1 and PRDX2 are integral members of the Peroxiredoxin (PRDX) family, pivotal in regulating cellular reactive oxygen species (ROS) metabolism (Table 4).^35^ Beyond the PRDX family, the Thioredoxin (TRX) family also contributes to cellular ROS metabolic pathways.^36^ Our identification of TXNDC17, which houses a Thioredoxin domain, hints at its potential role as a celastrol-binding protein (Table S2). These findings offer insights into previous studies of celastrol’s capacity to elevate ROS levels in cancer cells,^12^ and provide valuable hints for future studies aimed at elucidating the mechanisms by which celastrol modulates ROS levels in cancer cells.

**Table 4:** Annotations of Identified Proteins Between Groups Were from Uniprot

Spot no.	Protein identify	Biological process	Cellular component	Molecular function
**4**	Stress-induced-phosphoprotein 1 (STIP1)	Cellular response to interleukin-7	Nucleus, Cytoplasm	A cochaperone of heat shock proteins
**9 and 64**	Eukaryotic elongation factor 2 (EEF2)	Translational elongation	Nucleus, Cytoplasm	GTP-binding translation elongation factor family
**38**	Protein SETSIP (SETSIP)	Transcriptional activator	Nucleus	Histone binding, Chromatin binding
**39**	Peroxiredoxin-2 (PRDX2)	Cell redox homeostasis	Cytoplasm	Thiol-specific peroxiredoxin of antioxidant enzymes
**104**	Filamin-A (FLNA)	Cell junction assembly	Cytoskeleton	Actin-binding protein
**132**	Thioredoxin domain-containing protein 17 (TXNDC17)	Tumor necrosis factor-mediated signaling pathway	Cytoplasm	Disulfide reductase
**184**	Glyceraldehyde-3-phosphate dehydrogenase (GAPDH)	Glycolytic processing	Nucleus, Cytoplasm	A key enzyme in glycolysis
**221**	Neutral alpha-glucosidase AB (GANAB)	N-glycan processing	ER, Golgi apparatus	Glucan 1,3-alpha-glucosidase activity

### Confirming Celastrol’s Potential Binding Proteins *In Vitro*

Our findings indicated that STIP1, EEF2, and GAPDH exhibited notable shifts in abundance ratios (>2) on two-dimensional gels (Table S1). Consequently, these candidates were prioritized for validation through Western blotting and ITDRF_CETSA‑PULSE_ analysis to ascertain their potential as celastrol target proteins. The results showed that STIP1 was detected with three fragments (∼55 to 50, and ∼40 kDa) following CETSA-PULSE (Figures 4A-D and S7A). Also, celastrol significantly increased the susceptibility of STIP1 to protease digestion, resulting in a notable decrease in the *T*_m_ values of both STIP1 and its three fragments (Tables 2 and 3). Specifically, when celastrol concentrations exceeded 80 μM, the relative intensity of STIP1, along with the ∼55 and ∼40 kDa fragments, exhibited a negative correlation with celastrol concentration, whereas the ∼50 kDa STIP1 fragment remained unaffected by varying celastrol concentrations (Figures 4E and S7B). Conversely, observations suggest a protective effect of celastrol on GAPDH, evidenced by an increase in residue levels upon incubation with celastrol (Figures 5A and S8A). Although incubation with celastrol did not significantly alter the *T*_m_ value of GAPDH, it protected GAPDH from protease digestion at physiological temperatures (Tables 2 and 3). We also observed that even after heating at 61 °C, GAPDH is detectable when the sample is incubated with more than 160 μM celastrol (Figures 5B and S8B). Notably, despite the melting curve of GAPDH exhibiting a shift (Figure 5A), the melting curve within the 37 kDa band in the SDS-PAGE results did not show significant changes (Figure 1D). This discrepancy may arise because the sample within the 37 kDa band in SDS-PAGE likely contains a mixture of proteins or fragments rather than exclusively GAPDH. This observation further validates the reliability of the CETSA-PULSE method as it can precisely identify ligand-specific target proteins.

**Figure 4:**
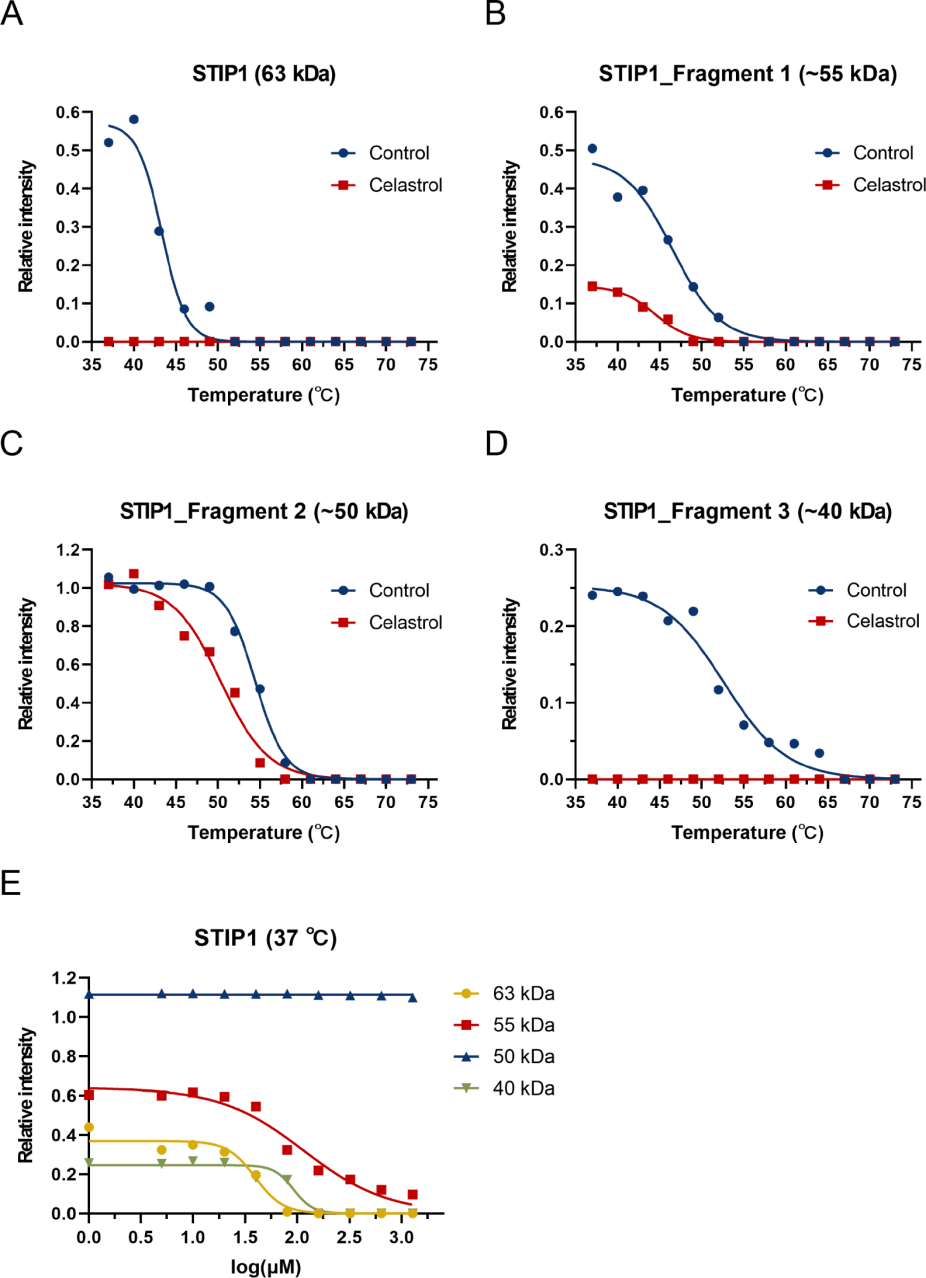
Confirming STIP1 as a celastrol potential binding target *in vitro*. (**A**) The melting curves of STIP1 (63 kDa), (**B**) STIP1 fragment 1 (∼55 kDa), (**C**) STIP1 fragment 2 (∼50 kDa), (**D**) and STIP1 fragment 3 (∼40 kDa) show thermal shifts between the two groups. The *R* ^*2*^ of the STIP1 (63 kDa) control group is 0.9748, and that of the celastrol group is 1. The *R* ^*2*^ of the STIP1 fragment 1 (∼55 kDa) control group is 0.9849, and that of the celastrol group is 0.9886. The *R* ^*2*^ of the STIP1 fragment 2 (∼50 kDa) control group is 0.9970, and the celastrol group is 0.9846. The *R* ^*2*^ of STIP1 fragment 3 (∼45 kDa) control group is 0.9742, and celastrol group is 1. (**E**) Dose–response curves (63–55, ∼50, and ∼40 kDa) show incubation samples with celastrol reduce STIP1 levels at 37 °C, although the ∼50 kDa curve shows a slight decrease. The *R* ^*2*^ of STIP1 (63 kDa) is 0.9707, that of STIP1 fragment 1 (∼55 kDa) is 0.9636, and that of STIP1 fragment 3 (∼45 kDa) is 0.9663. The *R* ^*2*^ of STIP1 fragment 2 (∼50 kDa) cannot be calculated.

**Figure 5:**
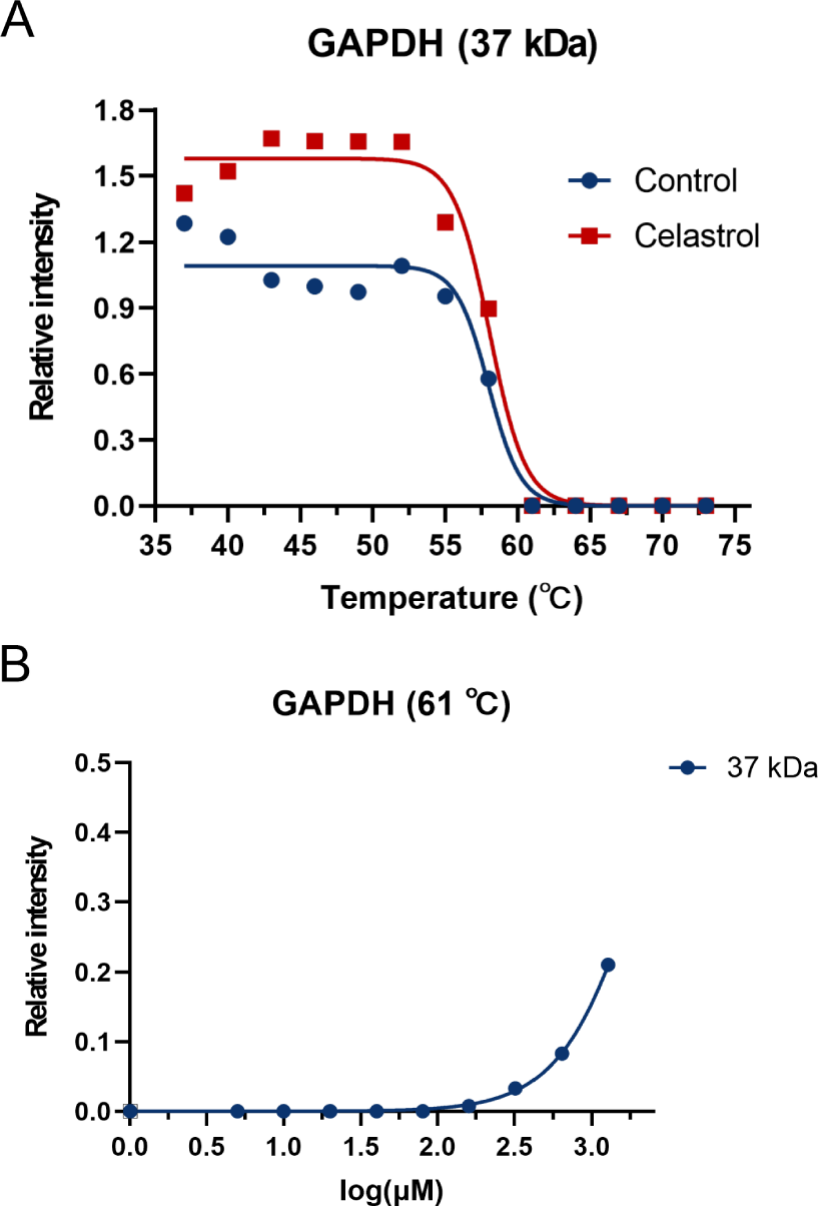
Confirming GAPDH as a celastrol potential binding target *in vitro*. (**A**) The melting curves of GAPDH (37 kDa) show thermal shifts between the two groups. The *R* ^*2*^ of the GAPDH (37 kDa) control group is 0.9735, and that of the celastrol group is 0.9846. (**B**) Dose–response curves (37 kDa) show that incubation of samples with high concentrations of celastrol increases GAPDH levels at 61 °C. The *R* ^*2*^ value of GAPDH (37 kDa) is 0.9993.

Previous studies have reported that celastrol can inhibit metastasis, which involves numerous abilities such as invasion and migration in various cancers.^37–40^ It is still unclear how celastrol suppresses tumor metastasis due to its complexity and association with multiple molecular pathways.^41^ Notably, three potential binding targets (GAPDH, FLNA, and STIP1) for celastrol indicated by our study may offer novel insights for this (Table 4). GAPDH is reported to have a higher expression level in CRC than normal tissue.^42^ Although GAPDH was originally considered a glycolytic enzyme, more and more studies pointed out that it is also involved in various biological functions, such as cellular endocytosis, cytoskeletal organization, iron metabolism, regulating some gene expression, and etc.^43,44^ Current research showed that through knockdown, its expression would affect CRC promotion and metastasis.^45^ Similarly, knockdown of FLNA was also found to significantly inhibit the metastasis of HCT116R cells in a mouse model.^46^ FLNA is one of the key proteins that promote CRC chemotherapy resistance in recent studies, and its high expression in CRC is correlated with the low survival rate of patients.^46^ Among the candidates we identified, STIP1 is also implicated in metastasis and has many biological functions.^47^ For instance, form a complex with hsp70/90 to regulate the degradation and folding of proteins,^48^ or be secreted out of cells to influence cell proliferation or apoptosis.^49^ To sum up, GAPDH, FLNA, and STIP1 have been confirmed to be related to cancer metastasis, and their multifunctionality is also important for maintaining cells (Table 4). Thus, we speculate that binding to these proteins may provide celastrol with multiple anticancer properties, including antimetastasis. Our results can provide a hint to investigate whether celastrol exerts its antimetastatic effects through these target proteins by examining its interactions with GAPDH, FLNA, and STIP1 in further studies.

Notably, the impact on EEF2 was distinct, with celastrol seemingly shielding EEF2 and a specific fragment (∼37 kDa) from proteolytic activity while reducing the remaining fragment (Figures 6A-C and S9A), and both I_37_ values exhibited a significant increase (Table 2). Conversely, in the control group, nearly all EEF2 was digested into fragments (∼30 kDa) following CETSA-PULSE at physiological temperatures. A potential conformational shift in EEF2 might render it less susceptible to Thermolysin, particularly noticeable when celastrol concentrations surpass 80 μM. This phenomenon likely contributes to the appearance of varied fragments postpulse-proteolysis (Figure 6D and Figure S9B). The primary spot representing EEF2 on the 2D gel is an ∼30 kDa fragment (Figure 3). Consequently, despite the negative abundance ratio in MALDI-TOF/TOF results (Table S1 and Figure 6A–C), celastrol appears to exert a protective effect on EEF2 against enzyme digestion.

**Figure 6:**
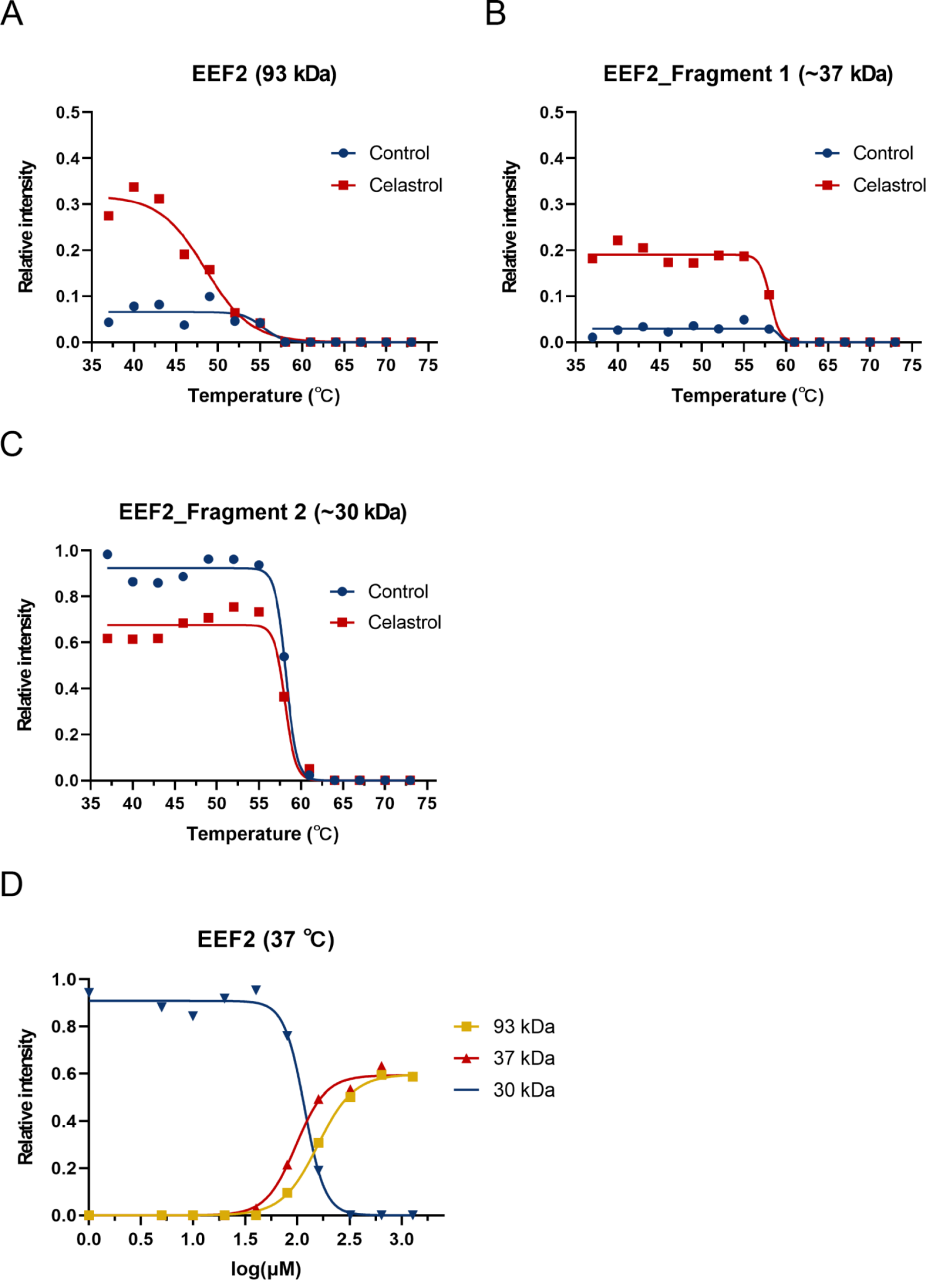
Confirming EEF2 as a celastrol potential binding target *in vitro*. (**A**) The melting curves of EEF2 (93 kDa), (**B**) EEF2 fragment 1 (∼37 kDa), and (**C**) EEF2 fragment 2 (∼30 kDa) show thermal shifts between the two groups. The *R* ^*2*^ of the EEF2 (93 kDa) control group is 0.7977, and that of the celastrol group is 0.9740. The *R* ^*2*^ of the EEF2 fragment 1 (∼37 kDa) control group is 0.7555, and that of the celastrol group is 0.9826. The *R* ^*2*^ of the EEF2 fragment 2 (∼30 kDa) control group is 0.9935, and the celastrol group is 0.9819. (**D**) Dose–response curves (93 and ∼37 kDa) show that incubation of samples with celastrol increases EEF2 levels but reduces EEF2 fragment 2 (∼30 kDa) levels at 37 °C. The *R* ^*2*^ of EEF2 (93 kDa) is 0.9988, EEF2 fragment 1 (∼37 kDa) is 0.9947, and EEF2 fragment 2 (∼30 kDa) is 0.9947.

From our findings, it is evident that CETSA-PULSE not only captures alterations in protein melting curves but also records changes in fragment residues across varying temperature points simultaneously. In Figure 6, the structural transformation of EEF2 upon incubation with celastrol seems apparent, leading to the detection of distinct fragments compared to the control group at identical temperature points. This observation potentially hints at configuration alterations upon binding of the compound to the protein, providing valuable insights for researchers. The implementation of ITDRF_CETSA‑PULSE_ enabled the observation of EEF2 fragment shifts induced by incubating different celastrol concentrations, offering guidance on the optimal dose for more effective target protein modulation (Figures 6D and S9B). Unlike classic CETSA, which primarily indicates protein–ligand binding targets, CETSA-PULSE suggests potential binding candidates and hints at conformational changes in proteins upon interaction with the compound.

EEF2 plays an important role in the process of translation and its function is related to ribosome translocation (Table 4). The level of EEF2 phosphorylation is closely related to its activity, which is known to be regulated by EEF2K.^50^ The elevated demand for swift protein synthesis in tumor cells potentially underpins the prevalent overexpression of EEF2 across various cancers,^51^ and the Human Protein Atlas also displays heightened EEF2 expression among CRC patients. Moreover, a previous study indicated that EEF2K is low expressed in around 80% of colorectal cancer patients, and downregulation of EEF2K was independently associated with poorer survival in colorectal cancer patients.^52^ Taken together, the inhibition of EEF2 may be an effective way to suppress cancer. Therefore, in future experiments we will continue to investigate how celastrol interacts with EEF2 and whether targeting it contributes to the inhibition of colon cancer.

In summary, the observed changes in the melting curves of EEF2, STIP1, and GAPDH strongly indicate specific interactions with celastrol, emphasizing the potential specificity of these interactions.

## Conclusion

In this study, we incorporated PULSE proteolysis into CETSA to improve the “nonspecific effects” that may occur with some ligands during the CETSA process. Similar to the classic CETSA, CETSA-PULSE identifies potential target proteins by monitoring the melting curve shift caused by the interaction between ligands and specific proteins. Therefore, it retains most of the advantages of CETSA, including the ability to simultaneously detect most proteins in samples, and it can be applied to *in vitro*, *in vivo*, or even tissue samples. In addition, because the protease digestion step is added to the CETSA-PULSE process, it can not only be used to monitor the shift of the melting curve but also detect possible configuration transformations when the protein interacts with ligands. Despite hydrolysis by Thermolysin possibly resulting in the loss of a few candidates, here, we still successfully identified eight potential binding targets through proteomic profiling integrated with CETSA-PULSE. We also confirmed GAPDH, FLNA, STIP1, and EEF2 as targets by Western blot and ITDRF_CETSA‑PULSE_. Despite the interactions between these potential target proteins and celastrol being preliminary and requiring additional mechanistic studies, these findings provide new insights into celastrol’s role in suppressing colon cancer.

Overall, CETSA-PULSE provides a new method to identify potential binding proteins for more ligands, and the target proteins that we identified by CETSA-PULSE can provide novel evidence to explain the mechanism of how celastrol suppresses cancer.

## Supplementary Material



## Data Availability

The complete data sets, including mass data, MALDI-TOF/TOF data, gel and western results, and analysis files, have been deposited in a public repository (Figshare, DOI: https://doi.org/10.6084/m9.figshare.28234742.v2). All data needed to evaluate the conclusions in the paper are present in the paper and/or the Supporting Information.

## Notes

Supplementary cytotoxicity of celastrol (Figure S1), CETSA-PULSE bioreplicates (Figure S2), CETSA results (Figure S3 and S4), 2D gel bioreplicates (Figure S5), Western blot figures (Figure S6–9), SAS analysis, MALDI-TOF/TOF Analysis and Mascot Search. Nine spots with significant difference by SAS analysis (Table S1). Comprehensive information on identified protein spots through MALDI-TOF/TOF Analysis and Mascot Search (Table S2). Experimental details for material and methods. Figure S1: The cytotoxicity of celastrol in the HCT116 cell line. Figure S2: Two other biological replicates for CETSA-PULSE results. Figure S3: Unveiling the potential binding targets of celastrol by CETSA. Figure S4: Two other biological replicates for CETSA results. Figure S5: Two other biological replicates for the 2D gel. Figure S6: Incubation samples with celastrol reduce FLNA levels. Figure S7: Incubation samples with celastrol reduce STIP1 levels. Figure S8: Incubation samples with celastrol reduce GAPDH levels. Figure S9: Incubation samples with celastrol reduce EEF2 levels. Table S1: Nine spots with significant difference by SAS analysis. Table S2: Comprehensive information on identified protein spots through MALDI-TOF/TOF Analysis and Mascot Search. Material and methods: The nanoLC gradient conditions. FlexAnalysis 3.0 software parameters for identified proteins of Gel-LC-MS/MS. IEF program for 2DE gel. Electrophoresis program for 2DE gel. Reflectron mode parameters of PMF (PDF)
