# Supplementary material for: Unveiling the Potential Binding Targets of Celastrol in Colorectal Cancer: A Proteomic Profiling Approach Integrating Cellular Thermal Shift Assay and Pulse Proteolysis
Source: J Proteome Res. 2025 Jun 6;24(7):3174–87. doi: 10.1021/acs.jproteome.4c00738 (PMC12235705; doi:10.1021/acs.jproteome.4c00738)
Supplement: Supplementary file 1 [file pr4c00738_si_001.pdf]

Supporting Information

Unveiling the Potential Binding Targets of Celastrol in Colorectal Cancer: A Proteomic Profiling Approach Integrating Cellular Thermal Shift Assay and Pulse Proteolysis

Authors:
Ti Lin[1,2¶], Shang-Lin Yang[1,3¶], Chao-Jung Chen[4], and Pei-Fen Liu[1*]

Affiliation:
[1] Department of Food Science and Biotechnology, National Chung Hsing University, Taichung City 402, Taiwan
[2] Graduate Institute of Microbiology, College of Medicine, National Taiwan University, Taipei 100, Taiwan
[3] Department of Biochemistry and Molecular Biology, National Cheng Kung University, Tainan City 701, Taiwan
[4] Graduate Institute of Integrated Medicine, China Medical University, Taichung City 402, Taiwan

Correspondence Author:
pfliu@dragon.nchu.edu.tw

Address:
Department of Food Science and Biotechnology, National Chung Hsing University, 145 Xingda Rd., South Dist., Taichung City 402, Taiwan

¶ These authors contributed equally to this work.

SUPPORTING INFORMATION

Supplementary cytotoxicity of celastrol (Figure S1), CETSA-PULSE bioreplicates (Figure S2), CETSA results (Figure S3 and 4), 2D gel bioreplicates (Figure S5), western blot figures (Figure S6-9), SAS analysis, MALDI-TOF/TOF Analysis and Mascot Search. Nine spots with significant difference by SAS analysis (Table S1). Comprehensive information on identified protein spots through MALDI-TOF/TOF Analysis and Mascot Search (Table S2). Experimental details for material and methods.

The following supporting information is available free of charge at ACS website http://pubs.acs.org.

Figures:

Figure S1. The cytotoxicity of celastrol in HCT116 cell line
Figure S2. Two other biological replicates for CETSA-PULSE results
Figure S3. Unveiling the potential binding targets of celastrol by CETSA
Figure S4. Two other biological replicates for CETSA results
Figure S5. Two other biological replicates for 2D gel
Figure S6. Incubation samples with celastrol reduce FLNA levels
Figure S7. Incubation samples with celastrol reduce STIP1 levels
Figure S8. Incubation samples with celastrol reduce GAPDH levels
Figure S9. Incubation samples with celastrol reduce EEF2 levels

Tables:

Table S1. Nine spots with significant difference by SAS analysis
Table S2. Comprehensive information on identified protein spots through MALDI-TOF/TOF Analysis and Mascot Search

Material and methods:

The nanoLC gradient conditions
FlexAnalysis 3.0 software parameters for identified proteins of Gel-LC-MS/MS
IEF program for 2DE gel
Electrophoresis program for 2DE gel
Reflectron mode parameters of PMF

Supplementary Figure 1

A

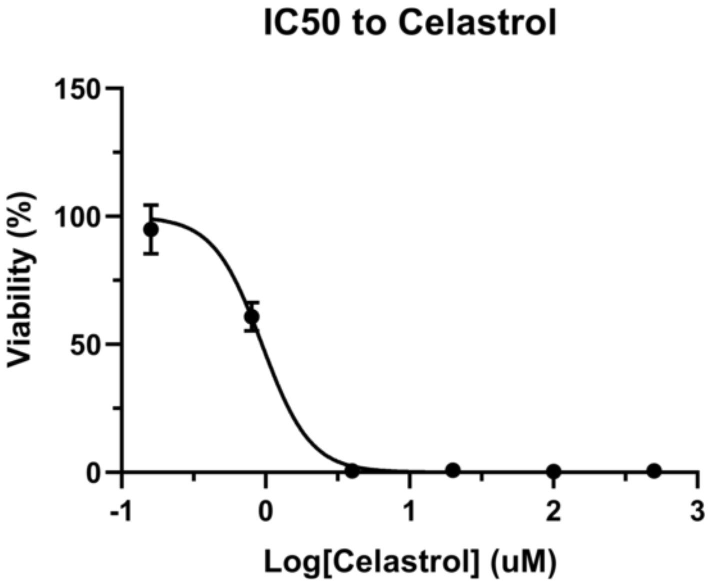

Compound	IC50 (HCT116)
Celastrol (CEL)	0.943 ± 0.087(μM)

Supplementary Figure 1. The cytotoxicity of celastrol in HCT116 cell line.

Supplementary Figure 2

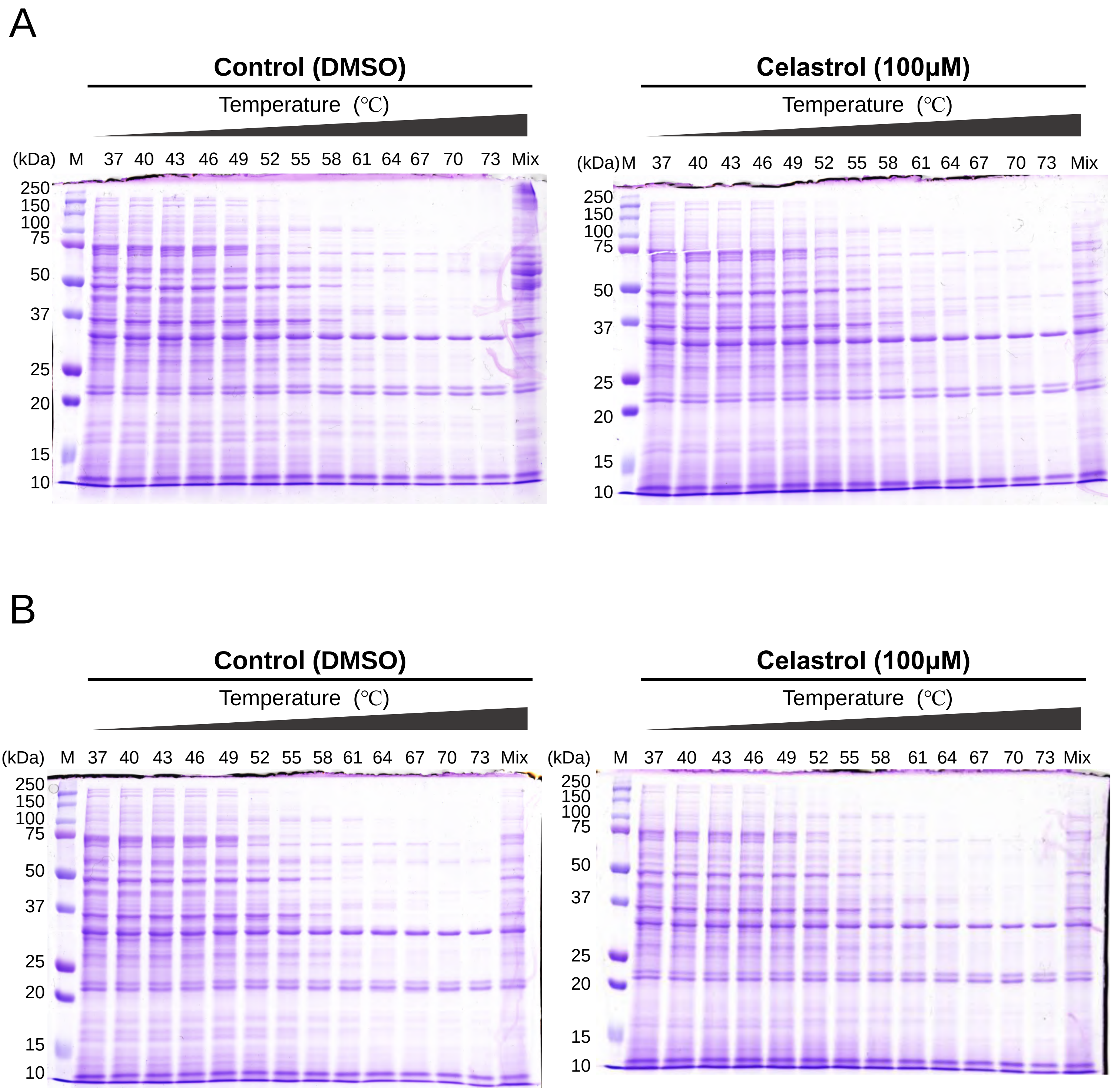

Supplementary Figure 2. Two other biological replicates **(A)** bioreplicate #2 and **(B)** bioreplicate #3 for CETSA-PULSE results. The other replicate is shown in **Figure 1B.**

Supplementary Figure 3

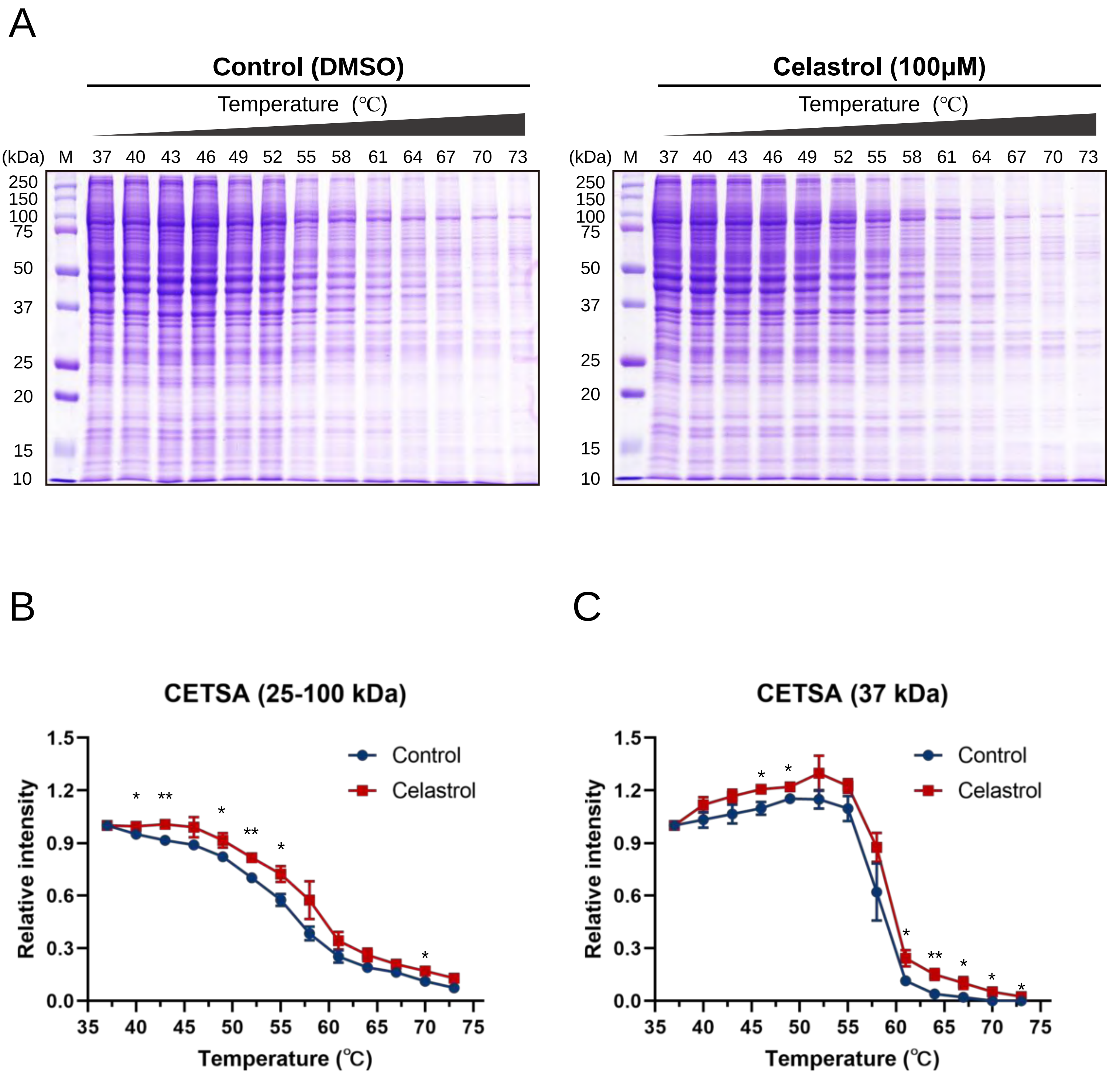

Supplementary Figure 3. Unveiling the potential binding targets of celastrol by CETSA. **(A)** The samples of a control group (DMSO) and the experimental group (100 µM celastrol) were detected by SDS-PAGE, and the 25-100 kDa and 37 kDa bands were quantified. The other replicates are shown in **Supplementary Figure 4.** The melting curves of bands (**(B)** 25-100 kDa and **(C)** 37 kDa) show the significant thermal shifts between the two groups. Result are plotted as average ± S.D., *$p < 0.05$, **$p < 0.01$, ***$p < 0.001$ using an unpaired two-tailed Student's t-test.

Supplementary Figure 4

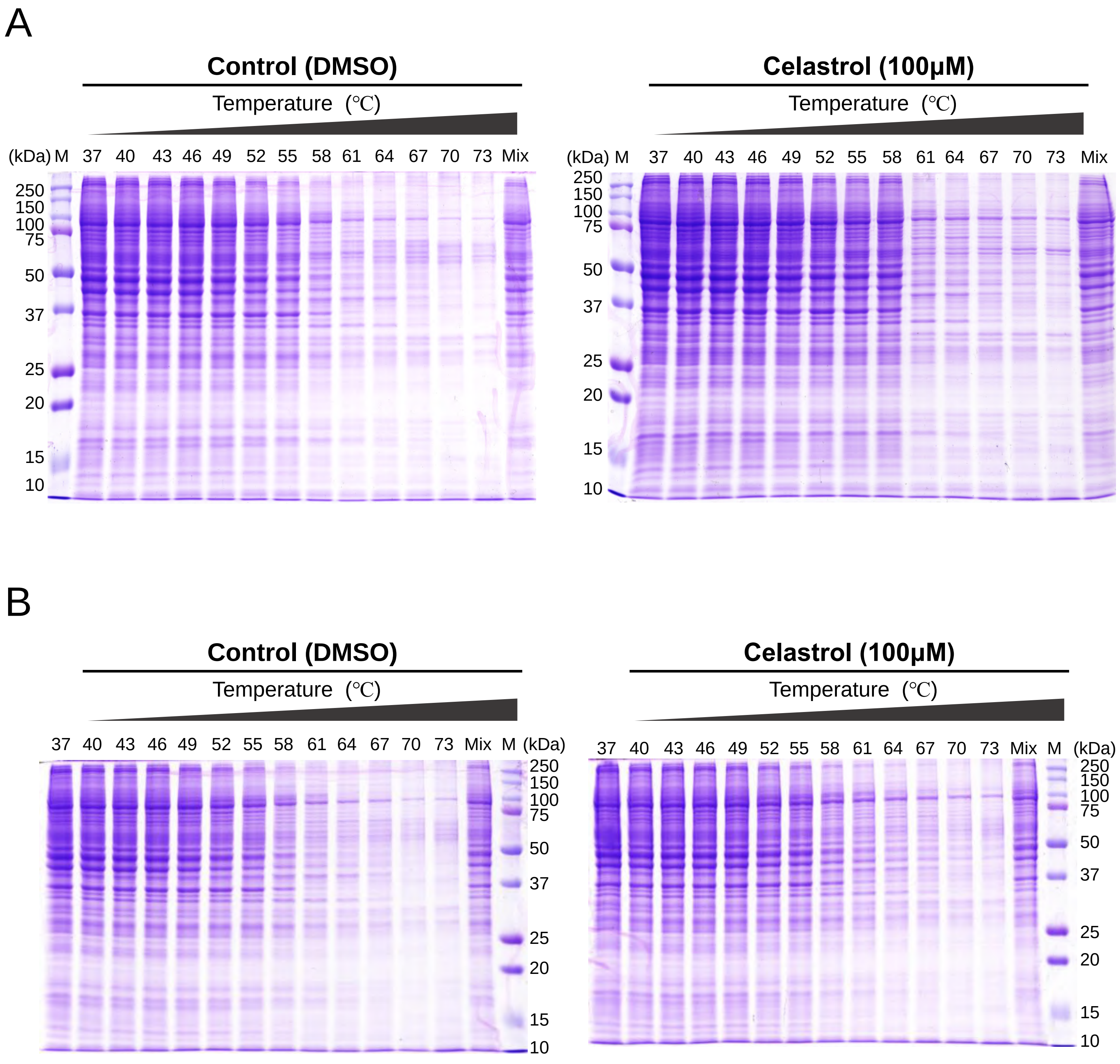

Supplementary Figure 4. Two other biological replicates **(A)** bioreplicate #2 and **(B)** bioreplicate #3 for CETSA results. The other replicate is shown in **Supplementary Figure 3.**

Supplementary Figure 5

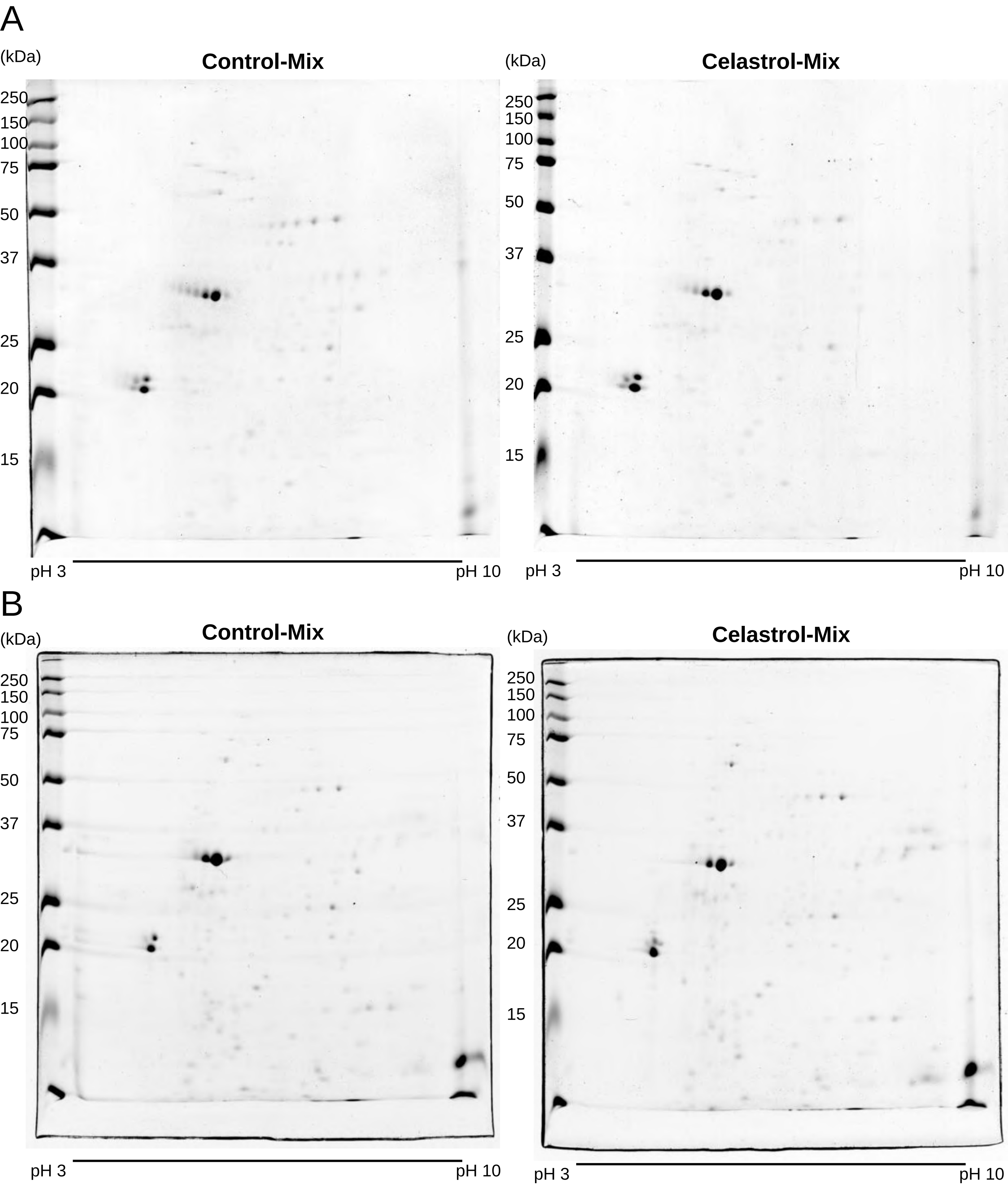

Supplementary Figure 5. Two other biological replicates **(A)** bioreplicate #2 and **(B)** bioreplicate #3 for 2D gel. The other replicate is shown in **Figure 3**.

Supplementary Figure 6

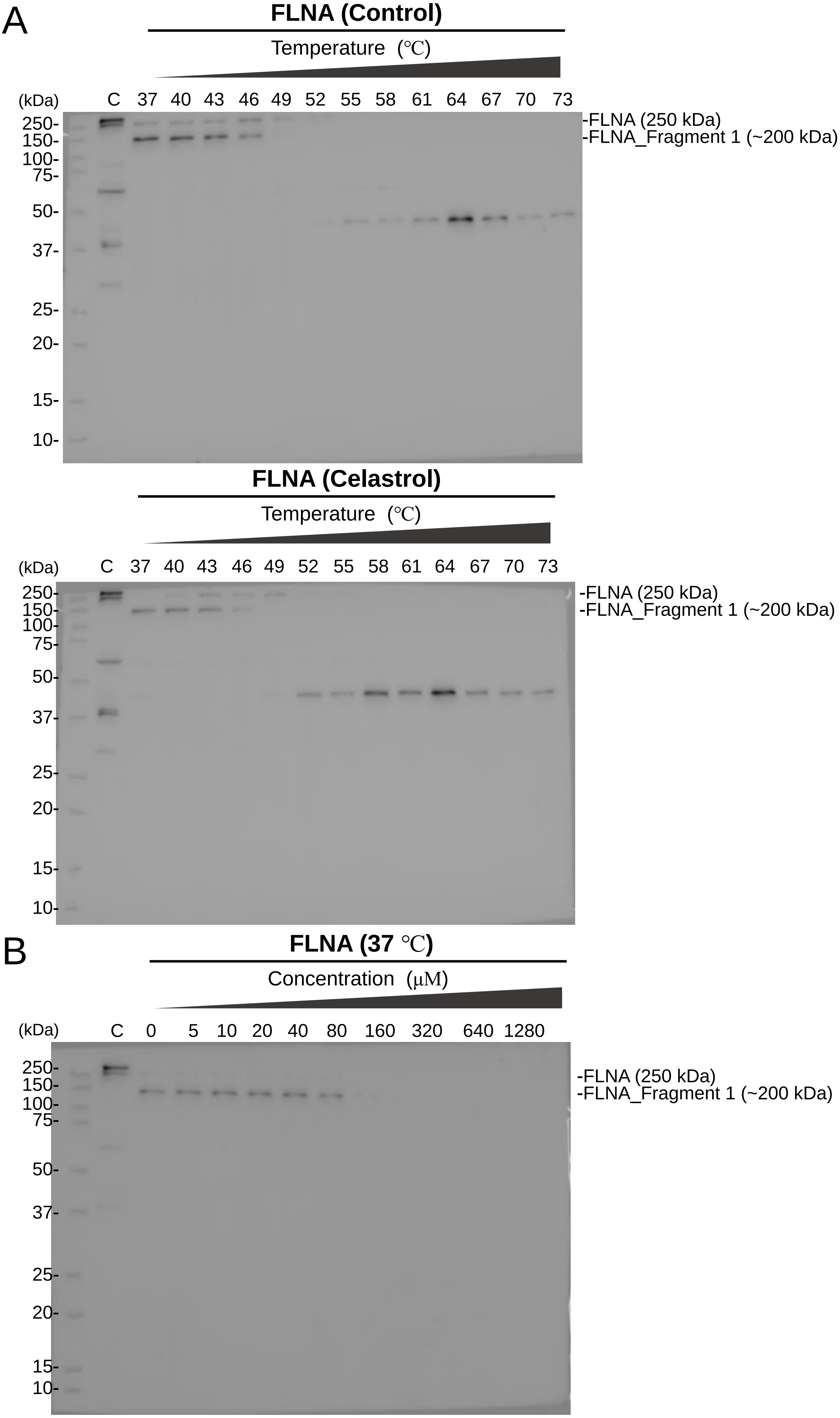

Supplementary Figure 6. Incubation samples with celastrol reduce FLNA levels. The protein level of FLNA was analyzed by western blot. **(A)** Effect of celastrol on FLNA (250 kDa) and its fragment (~200 kDa) levels in the same temperature gradient. **(B)** Effect of celastrol concentration gradient on FLNA (250 kDa) and its fragment (~200 kDa) levels at 37 °C.

Supplementary Figure 7

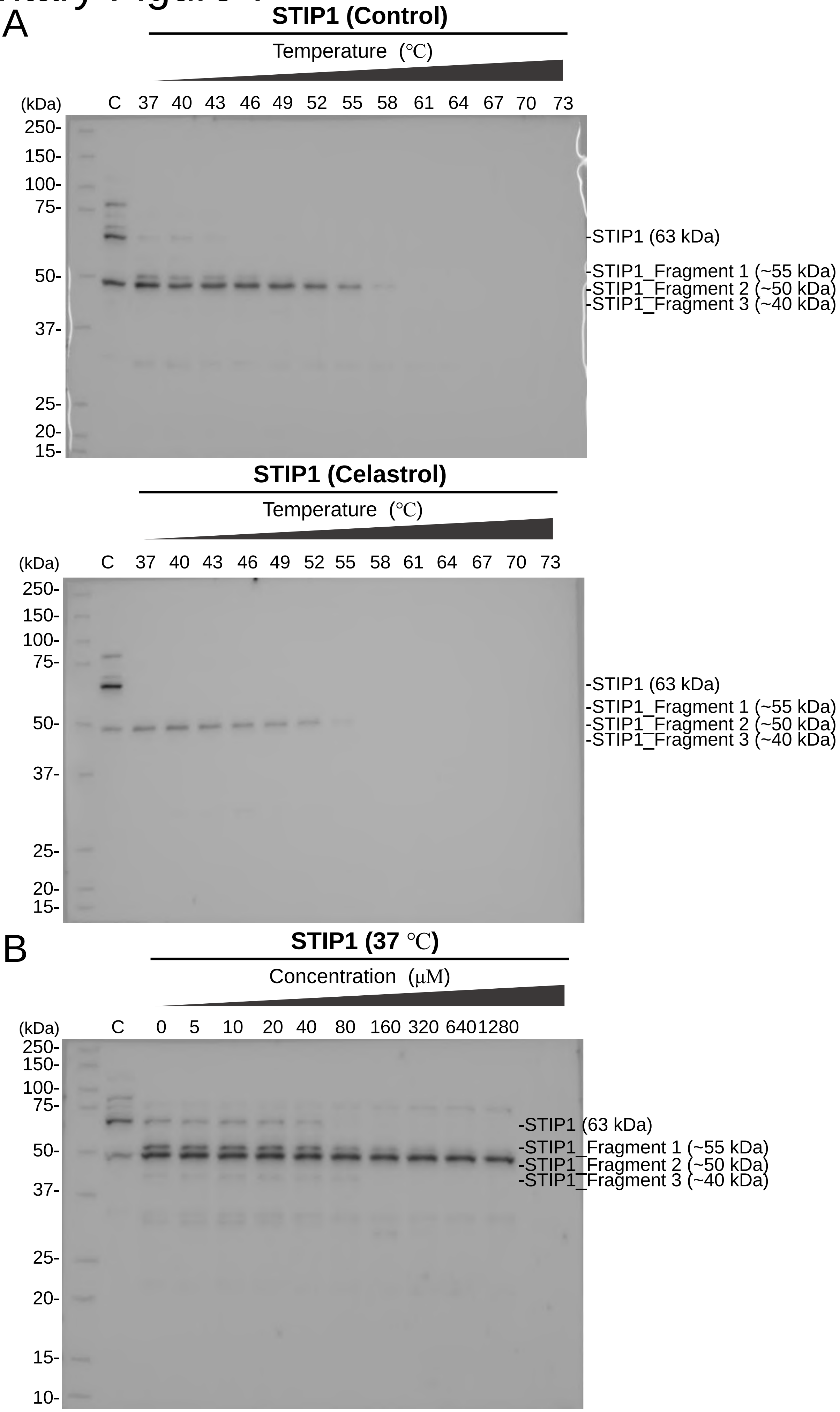

Supplementary Figure 7. Incubation samples with celastrol reduce STIP1 levels. The protein level of STIP1 was analyzed by western blot. **(A)** Effect of celastrol on STIP1 (63 kDa) and its fragments (~55 kDa, ~50 kDa, and ~40 kDa) levels in the same temperature gradient. **(B)** Effect of celastrol concentration gradient on STIP1 (63 kDa) and its fragments (~55 kDa, ~50 kDa, and ~40 kDa) levels at 37 °C.

Supplementary Figure 8

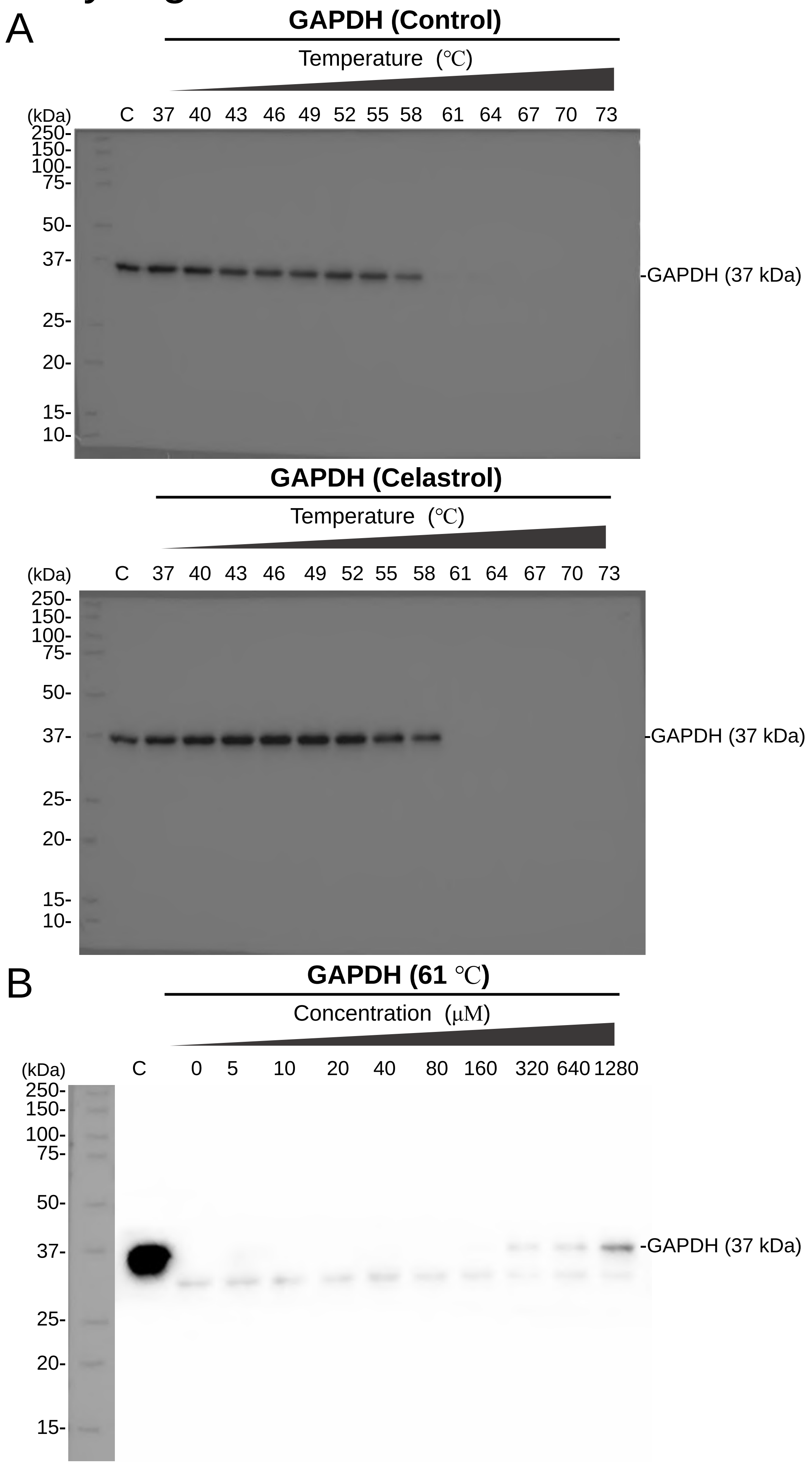

Supplementary Figure 8. Incubation samples with celastrol reduce GAPDH levels. The protein level of GAPDH was analyzed by western blot. **(A)** Effect of celastrol on GAPDH (37 kDa) levels in the same temperature gradient. **(B)** Effect of celastrol concentration gradient on GAPDH (37 kDa) levels at 61 °C.

Supplementary Figure 9

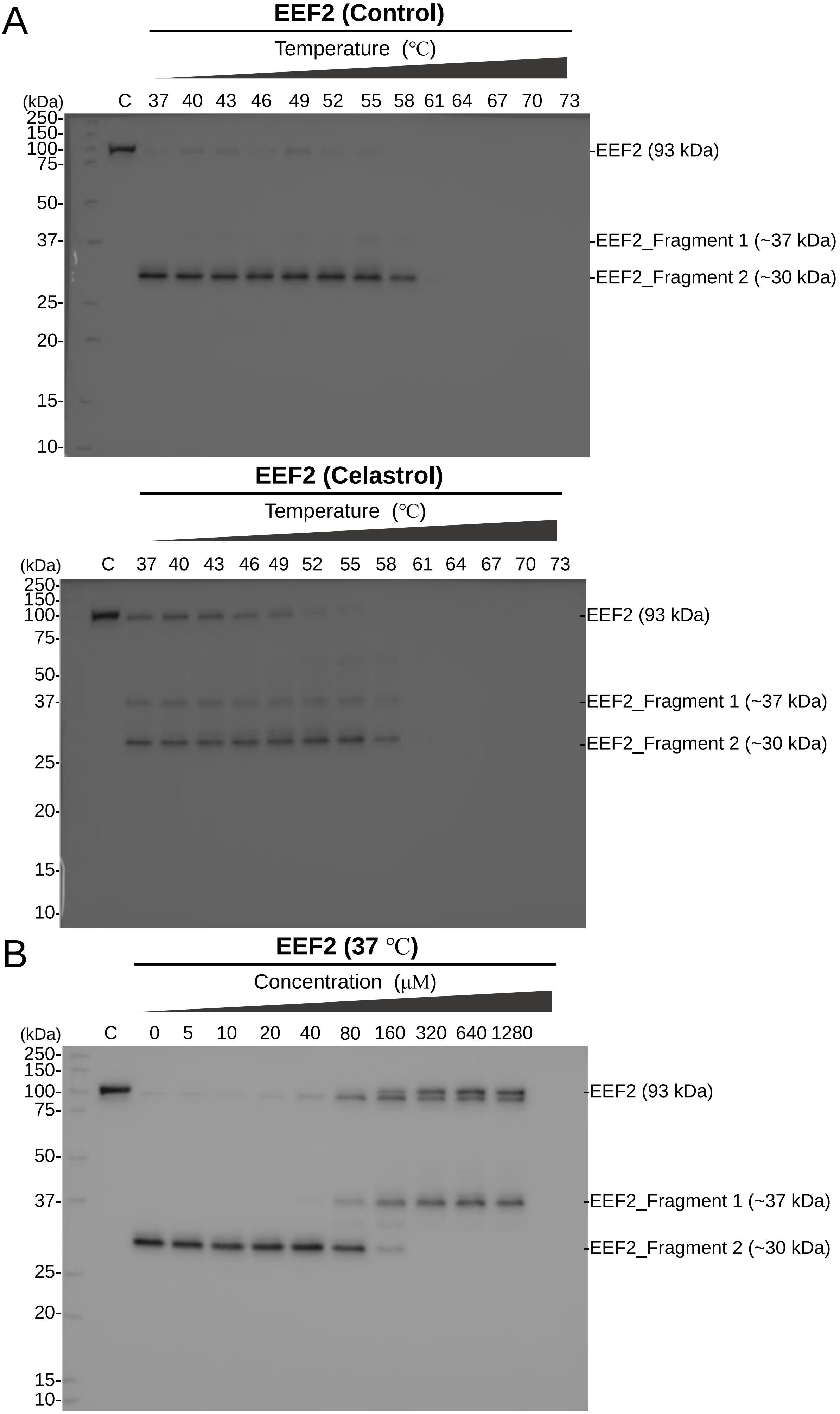

Supplementary Figure 9. Incubation samples with celastrol reduce EEF2 levels. The protein level of EEF2 was analyzed by western blot. **(A)** Effect of celastrol on EEF2 (93 kDa) and its fragments (~37 kDa and ~30 kDa) levels in the same temperature gradient. **(B)** Effect of celastrol concentration gradient on EEF2 (93 kDa) and its fragments (~37 kDa and ~30 kDa) levels at 37 °C.

Supplementary Table 1. Nine spots with significant difference by SAS analysis.

Spot number[1]	Treatment[2]		Abundance ratio[3]
	Control	Celastrol	
4(p=0.048)	0.4049 ± 0.0851a	0.1925 ± 0.0985b	-2.1034
9(p=0.014)	1.1125 ± 0.2871a	0.3452 ± 0.1385b	-3.2231
38(p=0.034)	0.2281 ± 0.0383a	0.1204 ± 0.0451b	-1.8939
39(p=0.016)	0.0754 ± 0.0123b	0.1041 ± 0.0011a	+1.3815
64(p=0.049)	0.4420 ± 0.1427a	0.2118 ± 0.0104b	-2.0872
104(p=0.005)	0.0630 ± 0.0091a	0.0327 ± 0.0025b	-1.9283
132(p=0.012)	0.1081 ± 0.0102b	0.1554 ± 0.0155a	+1.4366
184(p=0.026)	0.0916 ± 0.0101b	0.1869 ± 0.0047a	+2.0404
221(p=0.037)	0.0525 ± 0.0083a	0.0354 ± 0.0048b	-1.4827

Note:

1. The numbers of protein spots were named by Melanie 8.
2. This column means the relative volume of each spots to the total volume of all quantified spots in one gel. The superscripts (a or b) in same row indicate the significant difference between groups. ($p < 0.05$) Values are shown as mean±SD. (n = 3)
3. When the expression ratio reaches to 1.3, it indicates the significant difference between groups.

Supplementary Table 2. Comprehensive information on identified protein spots through MALDI-TOF/TOF Analysis and Mascot Search.

Spot no.[1]	Protein identity	Gene symbol	GenBank accession[2]	Theoretical (Mr / pI)	MALDI-MS PMF[3]	Score / Threshold[4]	
						TOF	TOF/TOF
**4**	Stress-induced-phosphoprotein 1	STIP1	12804257	63227/6.4	27/124(42)	111/56	148/29
**9**	Eukaryotic elongation factor 2	EEF2	4503483	96246/6.4	23/118(28)	98/56	361/28
**38**	Protein SETSIP	SETSIP	567757558	34861/4.0	9/98(28)	100/56	354/26
**39**	Peroxiredoxin-2	PRDX2	24659879	22049/5.6	11/115(42)	110/56	376/28
**64**	Eukaryotic elongation factor 2	EEF2	4503483	96246/6.4	32/152(35)	130/56	354/28
**104**	Filamin-A	FLNA	326205158	283301/5.7	19/47(12)	76/56	40/29
**132**	Thioredoxin domain-containing protein 17	TXNDC17	14249348	14217/5.3	NS[5]/93(22)	NS[5]	175/27
**184**	Glyceraldehyde-3-phosphate dehydrogenase	GAPDH	53734502	36201/9.3	11/73(46)	99/56	203/28
**221**	Neutral alpha-glucosidase AB	GANAB	66346737	63227/5.7	22/123(23)	106/56	168/29

Note:

1. The spot numbers represent the same name on 2D electrophoresis gels.
2. Accession numbers are based on NCBI database.
3. PMF is the number of matched peptides resulting from MALDI-MS PMF analysis. Sequence coverage presented in brackets means the ratio of portion sequence covered by matched peptide to the full length of the protein sequence.
4. The score refers to the results of MALDI-TOF and MALDI-TOF/TOF analysis with the MASCOT search engine.
5. NS represents a protein with no significant match in the database.

Material and methods

The nanoLC gradient conditions

The nanoLC gradient conditions were as follows: 10% to 40% (v/v) buffer B (80%ACN/0.1% FA) for 41 minutes, and then to 99% B for 0.1 minutes; hold at 99% B for 3.9 min, then return to 90% buffer A (2%ACN and 0.1% FA) for 11 min. Eight precursors of charge +2, +3, and +4 from each TOF MS scan were dynamically selected and isolated for MS/MS fragment ion scanning. The selected precursors were then actively excluded for 15 seconds. The MS and MS/MS accumulation were set at 1 and 4 Hz, respectively.

FlexAnalysis 3.0 software parameters for identified proteins of Gel-LC-MS/MS

The following search parameters were used: enzyme-trypsin, taxonomy-Homo sapiens, Fixed modifications-Carbamidomethyl (C), Variable modifications-Oxidation (M), mass tolerance for the monoisotopic peptide window was set to ±50 ppm, max missed cleavage: 2, Peptide Charge State: 1+,2+,3+. An identification is accepted based on a significant Mascot score ($p < 0.05$).

IEF program for 2DE gel

IEF was programmed as follows: (1) 30 V, 12 h; (2) 200 V, 1 h; (3) 500 V, 1 h; (4) 1000 V, 1 h; (5) 10000 V, 1h; (6) 10000 V, 1h; (7) 10000 V, 1h; (8) 10000 V, 48000 V/h and maintained at the voltage 500 V.

Electrophoresis conditions for 2DE gel

Electrophoresis was carried out at 4 °C with two steps: 600 V,120 mA, 2.5 W/gel, 20 min; 600 V, 300 mA, 6W/gel, 3 h. 2D gels were stained with Coomassie Brilliant Blue G-250, and the images were scanned using ImageScanner (GE Healthcare).

Reflectron mode parameters of PMF

Reflectron mode parameters of PMF are as follows: accelerating voltage: 25.0 kV, reflectron voltage: 26.3kV, pulsed ion extraction time: 80 ns, laser intensity: 13%, laser gun number: 750 shots, detection range: 1100-3680 m/z.

FlexAnalysis 3.0 software parameters for identified proteins of MALDI-TOF/TOF

The following search parameters were used: enzyme-trypsin, taxonomy-Homo sapiens, Fixed modifications- Carbamidomethyl (C), Variable modifications-Deamidated (NQ) and Oxidation (M), Mass values: Monoisotopic, Protein Mass:

Unrestricted, Peptide Mass Tolerance: ±100 ppm, Peptide Charge State:1+, Max Missed Cleavages: 1. an identification is accepted based on a significant Mascot score ($p < 0.05$).
